# Opioids and the Risk of Fracture: A Self-Controlled Case Series Study in the Clinical Practice Research Datalink

**DOI:** 10.1093/aje/kwab042

**Published:** 2021-02-19

**Authors:** Emily J Peach, Fiona A Pearce, Jack Gibson, Andrew J Cooper, Li-Chia Chen, Roger D Knaggs

**Keywords:** bone fractures, opioid analgesics, pharmacoepidemiology, self-controlled case series

## Abstract

Self-controlled study designs can be used to assess the association between exposures and acute outcomes while controlling for important confounders. Using routinely collected health data, a self-controlled case series design was used to investigate the association between opioid use and bone fractures in 2008–2017 among adults registered in the United Kingdom Clinical Practice Research Datalink. The relative incidence of fracture was estimated, comparing periods when these adults were exposed and unexposed to opioids, adjusted for time-varying confounders. Of 539,369 people prescribed opioids, 67,622 sustained fractures and were included in this study. The risk of fracture was significantly increased when the patient was exposed to opioids, with an adjusted incidence rate ratio of 3.93 (95% confidence interval (CI): 3.82, 4.04). Fracture risk was greatest in the first week of starting opioid use (adjusted incidence rate ratio: 7.81, 95% CI: 7.40, 8.25) and declined with increasing duration of use. Restarting opioid use after a gap in exposure significantly increased fracture risk (adjusted incidence rate ratio: 5.05, 95% CI: 4.83, 5.29) when compared with nonuse. These findings highlight the importance of raising awareness of fractures among patients at opioid initiation and demonstrate the utility of self-controlled methods for pharmacoepidemiologic research.

## Abbreviations

aIRR: adjusted incidence rate ratio
CI: confidence interval
CPRD: Clinical Practice Research Datalink
IRR: incidence rate ratio
OMEQ: oral morphine equivalent
SCCS: self-controlled case series

Fractures are a global public health concern; there are approximately 8.9 million osteoporotic fractures worldwide each year (1). Opioids may increase the risk of fracture, because of acute central nervous system effects, which include sedation and dizziness, and potential long-term effects on bone mineral density (2). An increased risk of fracture has been reported for users of opioids (3–5); however, methods were used in these studies to statistically match opioid users to nonusers to make comparisons in fracture risk possible; consequently, these studies are limited by the high potential for confounding.

In this study, we assessed the association between opioid use and fractures. We investigated the association between the duration and dose of opioid exposure and the risk of fracture by using a self-controlled study design to minimize confounding.

## METHODS

### Data source

We used data from patients registered with general practices in the United Kingdom that contribute data to the Clinical Practice Research Datalink (CPRD) GOLD. The CPRD GOLD is 1 of the largest databases of anonymized electronic health records, containing, among other routine health data, demographic information, prescription records, and medical diagnoses for more than 17 million individuals. In addition, the CPRD GOLD provides linkage to Hospital Episode Statistics, an administrative database that contains hospital records for English patients. Data access was approved by the Independent Scientific Advisory Committee of the Medicines and Healthcare products Regulatory Agency (protocol reference 18_282R).

### Study design

We used the self-controlled case series (SCCS) study design, which has been used in previous pharmacoepidemiologic studies to investigate fractures associated with thiazolidinediones (6) and antidepressants, (7) as well as to study the association between opioid use and road traffic accidents (8).

In the SCCS design, all individuals experience the exposure and outcome of interest. Within-person comparisons are made by deriving an incidence rate ratio comparing the rate of the outcome when exposed to the unexposed rate. Individuals, therefore, act as their own control, with the major advantage that factors remaining constant within a person (e.g., genetic factors), including those that are unknown or unmeasured, are inherently controlled for by design.

### Selection of cases

The base cohort, from which the SCCS cohort was selected, included individuals aged 18 years or older who started using opioids during the 9-year study period (June 1, 2008, to May 31, 2017). Patients entered the study 2 years after the observation start date, which was the latest of the following: the date of practice registration, the date the practice provided research-quality data, or June 1, 2006. The study exit date was the earliest of the following: the date of deregistration from the practice, the date the practice ceased to provide data to the CPRD GOLD, the date the patient died, or the study end date (May 31, 2017).

**Figure 1:**
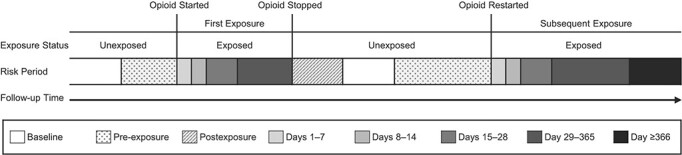
Division of exposed and unexposed follow-up time into risk periods. Fractures occurring in pre-exposure and postexposure periods are treated as neither unexposed time nor exposed time in the analysis.

Patients were excluded from the base cohort if they were prescribed an opioid in the 2 years between their observation start date and study entry date, if they sustained a fracture in the 6 months before their study entry date, or if they had a record of a fracture with a missing date. Cases included in the SCCS cohort were those recorded as having sustained at least 1 fracture during follow-up.

### Outcome

Fractures were identified using clinical codes for diagnoses (Web Table 1) (available at https://doi.org/10.1093/aje/kwab042), operations, and procedures that were recorded in the CPRD GOLD and Hospital Episode Statistics databases. If a patient had more than 1 record of a fracture, the earliest record was considered the first fracture. Subsequent fracture records were assumed to be new if they occurred in a different anatomical site or were recorded more than 6 months after a preceding fracture to the same site. If not, these fracture records were considered to relate to the earlier fracture records and were excluded from the analysis.

### Exposures

Exposure to opioids on a given day of follow-up was based on the presence of a prescription for an opioid analgesic (Web Table 2). We used an approach adapted from Pye et al., (9) which systematically handles missing data and prepares prescription records for time-varying analysis (Web Figure 1) (available at https://doi.org/10.1093/aje/kwab042) to generate a time-varying measure of opioid exposure. Consecutive prescriptions for identical opioid products were combined into 1 episode, allowing for a permissible gap of 15 days. The prescription duration (in days) was calculated on the basis of the prescribed daily dose and quantity prescribed. The stop date for each prescription record was calculated using the prescription start date and duration. Clinical equianalgesic ratios were used to covert opioid doses to oral morphine equivalent (OMEQ) doses (milligrams/day), representing the analgesic potency of an opioid relative to oral morphine (Web Table 2). Any duplicate or overlapping prescriptions were combined to generate a binary indicator for exposure or nonexposure with an OMEQ dose on each day of follow-up.

Periods of exposure to opioids were split into discrete risk periods for the first period of exposure to opioids and any subsequent periods of opioid exposure. Risk periods reflected the proximity of opioid exposure to the date an opioid was started or re-started. Exposed risk periods consisted of days 1–7, 8–14, 15–28, 29–365, and day 366 until the final day of opioid exposure within that period, where day 1 refers to the day after a person began to take an opioid or re-started taking an opioid (Figure 1). To reduce protopathic bias, the date the opioid was started or re-started was not included in the exposed risk periods (10).

Periods of nonexposure to opioids consisted of all follow-up time before the date the opioid was started, during any gaps between exposed periods, and after the final exposed day until the date follow-up ended. A 90-day pre-exposure period was included to eliminate bias arising from event-dependent exposure (11). Fracture events and person-time occurring in the 90 days before, and including the day an opioid was started or restarted, were consequently removed from the baseline (i.e., unexposed) rate of fracture (Web Figure 2); the inclusion of these fracture events would have otherwise underestimated the risk of fracture when the individual was exposed to an opioid. A 28-day post-exposure period was introduced to reduce bias resulting from residual associations with opioids after cessation. Figure 1A illustrates the division of follow-up time into these discrete periods; the lengths of risk periods were curtailed if they overlapped with the start of a subsequent risk period (Web Figure 3).

### Confounding variables

The SCCS design inherently controls for unmeasured time-invariant and between-individual confounding; however, within-person factors that vary over time needed to be controlled for. After consideration of covariates included in similar studies (3–5) and of factors found to affect fracture rates (12–16), age, season, and exposure to fracture-risk increasing drugs (Web Table 3) were adjusted for in this analysis, providing they significantly improved the model fit (17). To adjust for time-varying confounders, each risk period was divided into smaller periods to account for changes in age (yearly), the season of year (*n* = 4 (3 months each)), and exposure to fracture risk–increasing drugs (binary indicator in 3-month intervals). Doing so allowed for an adjustment of fracture risk over time, because these covariates changed throughout follow-up.

### Statistical analysis

Fixed-effects Poisson regression models, conditioned on the individual, were used to estimate crude IRRs, adjusted IRRs (aIRRs), and 95% confidence intervals, comparing the rate of fracture when exposed with the baseline rate.

The decision of whether to fit age as a continuous or categorical variable was made by fitting age as both a continuous variable and as a categorical variable (at 1-year intervals) and running separate models with each. The likelihood ratio test was run to compare model fit in both instances, with the variable with the best fit being carried forward into the final model. In building the final model, all covariates were included in a model and their associations assessed by first running the model with all covaries and then removing just the 1 covariate under investigation and assessing model fit using the likelihood ratio test. If the model fit was significantly (*P* < 0.05) improved by including the covariate, then it was included in the final model. The advantage of taking a backward elimination approach is that the joint predictive ability of variables is assessed, leaving only the most important variables in the model. The results were stratified to consider associations by age group (<65, ≥65 years), sex (male, female), and OMEQ dose (<50 mg/day, ≥50 mg/day) to assess associations by dose. Additional investigative analyses explored age-sex and dose-duration interactions.

This study was part of a program of research that included all available patients from the CPRD GOLD to form the base cohort of patients exposed to opioids. Before this SCCS analysis, in a pre hoc sample-size calculation to determine study feasibility, we estimated the sample size required, using the signed root likelihood ratio formula (18). For this calculation, we used the median duration of observation for the base cohort, along with an IRR of 1.2, based on the results of prior opioid-fracture association studies. It was estimated that 26,953 fracture cases with a median observation period of 7.1 years were needed to detect a relative incidence of 1.2 within the first 28 days of exposure with 95% power and a 5% significance level. Statistical significance was set at 2-tailed *P* < 0.01. We used Stata/MP 15 (StataCorp LP, College Station, TX) for data management and statistical analyses.

### Sensitivity analyses

Individuals who died within 90 days of their first fracture were excluded to test the sensitivity of the results to the potential for fractures to influence the duration of observation. Fractures increase the risk of subsequent fractures (19); therefore, the analyses were carried out for first fractures only to test the sensitivity of the results to events that were not independent of each other. Bone metastases may increase fracture risk and the need for analgesia; thus, patients with a record of cancer were excluded to test for sensitivity to this potential confounding factor. The analyses were repeated for alternative durations of the pre-exposure; results from analyses using a 7- and 28-day pre-exposure period were compared with the 90-day pre-exposure period. A complete case analysis was performed to assess for potential bias arising from the handling of missing exposure data. The analyses were repeated for only fractures identified in the Hospital Episode Statistics database, because dates for events that require hospital admission may be more accurately recorded in Hospital Episode Statistics than in the CPRD GOLD database (20). In addition, fractures to some sites may be susceptible to delayed diagnosis; aIRRs were stratified by fracture site, and sites with aIRRs suggesting a more than 8-fold increase in fracture risk were excluded to test the sensitivity of the results to possible reverse causality. Finally, the principal analysis was repeated for falls as an outcome, because falls are likely to be a mediating factor between opioids and fractures.

## RESULTS

After applying the study exclusion criteria, the base cohort comprised 539,369 individuals (Web Figure 4). Of these, 67,622 individuals who sustained a total of 87,454 fractures and contributed a total of 452,347 person-years of follow-up were included in this SCCS study. Among these individuals, 58.7% (*n* = 39,677) were female; the mean age at study entry was 56.1 years (standard deviation, 19.6); 93.1% (*n* = 62,983) were White; 23.2% (*n* = 15,663) were from the least deprived areas; and the median duration of follow-up of was 7.1 (interquartile range, 5.3, 8.1) years (Table 1).

**Table 1:** Demographic Characteristics of People Included in a Self-Controlled Case Series Study of Opioid Use and Fractures (*n* = 67,622), United Kingdom, 2008–2017

**Variable**	**No.**	**%**
Duration of follow-up, years^a^	7.1 (5.3, 8.7)	
Age at index, years^b^	56.1 (19.6)	
Female sex	39,677	58.7
Index of multiple deprivation, quintile		
1 (least deprived)	15,663	23.2
2	14,903	22.0
3	13,934	20.6
4	12,235	18.1
5 (most deprived)	10,852	16.1
Missing	35	0.1
Ethnicity		
White	62,983	93.1
Asian or Asian British	1,137	1.7
Black or Black British	569	0.8
Other	447	0.7
Mixed	226	0.3
Unknown	2,260	3.3
Osteoporosis^c^	8,715	12.9
FRID during follow-up	42,463	62.8

Abbreviation: FRID, fracture-risk–increasing drug.

^a^ Values are expressed as median (interquartile range).

^b^ Values are expressed as mean (standard deviation).

^c^ Presence of an osteoporosis code at any time in the individual’s clinical data file.

### Associations with fracture

The crude IRR for fracture during the exposed risk period, relative to the baseline (unexposed) period, was 4.18 (95% CI: 4.07, 4.30). The likelihood ratio test indicated that the addition of age and season as covariates improved the model fit (*P* < 0.001), however, exposure to fracture-risk–increasing drugs did not significantly improve the model fit (*P* = 0.543); consequently, fracture-risk–increasing drugs were omitted from the adjusted analyses. After adjusting for age and season, the aIRR for the risk of fracture when exposed to opioids was 3.93 (95% CI: 3.82, 4.04) (Table 2).

**Table 2:** Incidence Rate Ratios for the Risk of Bone Fracture During Periods of Exposure to Opioids in a Self-Controlled Case Series Study (*n* = 67,622), United Kingdom, 2008–2017

**Risk Period**	**No. of Person-Years**	**Fractures**		**Unadjusted Model**		**Fully Adjusted Model** ^**a**^ ^,^ ^**b**^	
		**No.**	**%**	**IRR**	**95% CI**	**IRR**	**95% CI**
Baseline^c^	377,665	49,473	56.6	1.00	Referent	1.00	Referent
Pre-exposure	42,779	26,853	30.7	5.63	5.54, 5.72	5.49	5.40, 5.58
Postexposure	9,044	2,626	3.0	2.37	2.28, 2.47	2.31	2.22, 2.40
Exposed^d^	22,859	8,502	9.7	4.18	4.07, 4.30	3.93	3.82, 4.04
First							
Days 1–7	1,196	1,327	1.5	7.74	7.32, 8.17	7.81	7.40, 8.25
Days 8–14	828	592	0.7	5.03	4.64, 5.46	5.08	4.68, 5.51
Days 15–28	484	256	0.3	3.65	3.22, 4.13	3.65	3.23, 4.13
Day 29–365	846	219	0.3	1.81	1.58, 2.08	1.77	1.54, 2.03
Day ≥366	201	38	<0.1	1.44	0.99, 2.08	1.25	0.86, 1.82
Subsequent							
Days 1–7	4,248	2,080	2.4	5.45	5.20, 5.71	5.05	4.83, 5.29
Days 8–14	3,175	1,114	1.3	4.02	3.78, 4.27	3.72	3.50, 3.96
Days 15–28	2,788	823	0.9	3.42	3.18, 3.67	3.12	2.91, 3.36
Day 29–365	7,506	1,766	2.0	2.75	2.61, 2.91	2.43	2.30, 2.57
Day ≥366	1,587	287	0.3	2.18	1.91, 2.50	1.73	1.50, 1.98

Abbreviations: aIRR, adjusted incidence rate ratio; CI, confidence interval; IRR, incidence rate ratio.

^a^ All *P* values < 0.001.

^b^ IRR was adjusted for 1-year increments in age and 3-month intervals for season.

^c^ Baseline refers to any time an individual was not exposed to opioids (excluding the pre-exposure and postexposure periods).

^d^ Exposed refers to any time an individual was exposed to opioids.

After dividing exposed time into risk periods that corresponded to the duration of opioid use, the aIRR for fracture in days 1–7 of the first exposure period, compared with the baseline risk, was 7.81 (95% CI: 7.40, 8.25). The aIRRs steadily decreased as the duration of opioid use increased over the first exposure period (until a gap in exposure or cessation of opioids); the aIRR was 1.77 (95% CI: 1.54, 2.03) for days 29–365 and 1.25 (95% CI: 0.86, 1.82) for day 366 onward. The risk of fracture increased when opioids were restarted; the aIRR for days 1–7 of subsequent periods of exposure was 5.05 (95% CI: 4.83, 5.29), which decreased to 2.43 (95% CI: 2.30, 2.57) for days 29–365 and 1.73 (95% CI: 1.50, 1.98) for day 366 onward (Table 2).

When exploring associations by age, no difference was found; the aIRR for the risk of fracture when exposed to opioids was 3.76 (95% CI: 3.61, 3.91) for people younger than 65 years, and was 3.94 (95% CI: 3.79, 4.09) for people aged 65 years or older. After exploring associations by sex, men (aIRR = 4.15, 95% CI: 3.96, 4.35) had a greater risk of fracture when compared with women (aIRR = 3.55, 95% CI: 3.42, 3.69). No significant interaction was observed between age and sex.

**Figure 2:**
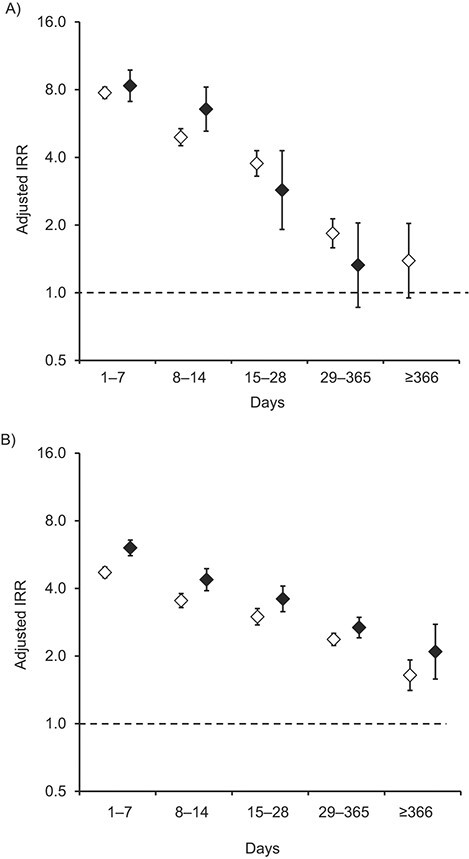
A) Incidence rate ratios (IRRs) for the risk of bone fracture during the first period of opioid exposure, by oral morphine equivalent (OMEQ) dose in a self-controlled case series study (*n* = 67,622), United Kingdom, 2008–2017. B) IRRs for the risk of bone fracture during subsequent periods of opioid exposure, by OMEQ dose in the self-controlled case series study. Hollow diamonds refer to adjusted IRRs for OMEQ doses <50 mg/day; black diamonds refer to adjusted IRRs for OMEQ doses ≥50 mg/day. There were insufficient data to estimate adjusted IRRs for fracture during days ≥366 of first exposures at OMEQ doses ≥50 mg/day. Incidence rate ratios were adjusted for 1-year increments in age and 3-month intervals for season. Values are plotted on a logarithmic scale.

To investigate associations by daily OMEQ dose, risk periods were stratified into periods of low (<50 mg/day) and high (≥50 mg/day) doses. The risk of fracture was greater when an individual was exposed to high daily doses of opioids (aIRR = 4.50, 95% CI: 4.26, 4.74) compared with low doses (aIRR = 3.90, 95% CI: 3.79, 4.02). When exploring the interaction between duration of opioid use and opioid dose, similar trends over time were found among periods of low-dose opioid use and high-dose opioid use. No significant interaction was observed between dose and duration of use after initiation of opioids (i.e., for the first period of opioid exposure), as shown in Figure 2A. A significant interaction was observed for periods after the restart of opioid use (i.e., for subsequent periods of exposure). The risk of fracture was greater in days 1–7 after the restart of opioid use for high (≥50 mg/day), compared with low (< 50 mg/day), OMEQ doses (aIRR = 6.06, 95% CI: 5.60, 6.56; and 4.71, 95% CI: 4.46, 4.98, respectively) (Figure 2B).

### Sensitivity analyses

The results from the sensitivity analyses did not considerably differ from the results presented in the primary analyses (Web Table 4). Fractures to the spine, chest, low back, and pelvis had greater aIRRs (Web Figure 5); after excluding these, aIRR values were slightly lower than the primary results (Web Figure 6). Opioid use was significantly associated with an increased risk of falls, which was greatest in the first week of opioid exposure; however, only a weak trend was observed when restricted to falls without fracture (Web Figure 7).

## DISCUSSION

This study is 1 of the largest and longest studies, and, to our knowledge, the first study using SCCS methodology, in which the association between opioids and fractures was investigated. There was nearly a 4-fold increase in the risk of fracture associated with periods of opioid exposure, compared with periods of nonuse. Furthermore, the risk of fracture was significantly greater when a person was exposed to opioids compared with periods of nonuse and was greatest (8-fold higher) during the initial week of use and when OMEQ doses were higher than 50 mg/day (6-fold higher) rather than 50 mg/day or less (4.7-fold higher), indicating both a duration- and dose-dependent association between opioid use and fractures.

The finding that opioid use increases the risk of fracture immediately after starting and restarting opioid use, and the finding of the magnitude of risk reported during these periods are novel. These findings support the hypothesized mechanism of action whereby opioids induce acute central nervous system effects, which result in a greater susceptibility to falls and fractures (2). This concept was further explored by investigating the association between opioids and falls not resulting in a fracture, which showed a lesser magnitude of association and a weak trend over time. One limitation with studying fall outcomes is the possibility that these falls may not require urgent medical attention and, therefore, may be less likely to be recorded in electronic health records. If they are reported, there may be a delay in doing so. The SCCS study design relies on having accurate dates for outcomes; therefore, a lack of precision in ascertaining falls may explain this finding. We also found in this study that although fracture risk declined with longer duration of opioid use, the risk of fracture remained elevated after 1 year of continuous opioid use. This finding warrants additional investigation of potential associations with bone mineral density.

### Comparison with other studies

Our findings are consistent with other opioid–fracture association studies that were conducted in populations outside of the United Kingdom (21–24), including a retrospective cohort study of 2,341 people in the United States, in which researchers found that people prescribed OMEQ doses of 50 mg/day or more had a higher risk of fracture (hazard ratio = 2.00, 95% CI: 1.24, 3.24) than those prescribed OMEQ doses of less than 20 mg/day (hazard ratio = 1.20, 95% CI: 0.92, 1.56), compared with people who were not using opioids (23). We found a greater risk of fracture was associated with higher opioid doses, although not in the first period of opioid exposure; very few people were initiated with high doses, which may explain the absence of a significant dose relationship in initial use.

Existing opioid–fracture association studies are susceptible to time-varying and time-invariant confounding as well as confounding by indication, making it difficult to establish whether the relationship might be 1 of cause and effect. We overcame many of the limitations of prior studies by adopting a self-controlled design for this study that circumvents issues of time-invariant unmeasured and between-individual confounding and limits potential confounding by indication. Given the significant positive association between opioid use and fractures reported in this and prior studies, there is compelling evidence for the existence of an association. Furthermore, we controlled for confounding to a greater extent than prior between-participant studies by design; thus our results suggest the confounding present in prior studies may have attenuated the magnitude of the association.

### Study strengths and limitations

Factors that vary over time are not inherently controlled for when using self-controlled methods. Although efforts were made to adjust for time-varying covariates, it is possible that some residual confounding remained, such as changes in lifestyle, muscle mass, body mass index, and pain condition, which were not well recorded. Nevertheless, the SCCS study design has the advantage of controlling for unmeasured time-invariant confounding, which cannot be controlled for by cohort and case–control designs (25). The target population consisted of people starting to take opioids; defining new use incorporating a 2-year lookback period does not guarantee these people were new users of opioids. There is also a potential of exposure misclassification, because it was assumed people had their opioid prescriptions dispensed and that they took them as indicated by the prescriber. People may have stopped taking their opioids, taken them differently than as prescribed, or obtained over-the-counter opioids via pharmacy purchases, hospitals, or illegitimate means, which would not have been recorded. Patients may have been exposed to opioids that were bought over the counter (i.e., codeine and paracetamol combinations available for purchase in the United Kingdom), which are not recorded in the CPRD GOLD database. Therefore, patient-time may have been classified as unexposed when, in this example, these patients may have been exposed to opioids. In addition, patients may not have taken opioids on days classified as exposed, because of the “when required” nature of these medicines; this may have led to misclassification of time as exposed when, in reality, patients were not exposed to opioids. It is not known in which direction exposure misclassification may have related to the timing of a fracture nor how this may have biased the results.

This study has several important strengths. The SCCS design relies on some assumptions, and violation of these can bias the results (11). There are 2 important assumptions: 1) events arise independently within individuals (i.e., fractures do not affect the occurrence of a subsequent fracture) and 2) events do not influence subsequent follow-up. These assumptions were tested for in sensitivity analyses by considering first fractures only and analyzing only patients who did not die within 90 days of fracture; the assumptions did not affect our results. In addition, fractures occurring on the first day of opioid exposure were incorporated into the pre-exposure risk-period incidence rate, which eliminated the introduction of protopathic bias, thereby reducing the likelihood of reverse causality. However, as a result, the risk of fracture on day 0 (i.e., the first day of opioid exposure) was not estimated, and this is expected to have resulted in an underestimation of the initial risk of fracture associated with opioid use. We defined incident fractures using the same definition as authors of a prior CPRD GOLD study of fractures (6). Although the definition used could have potentially under- or overestimated the incidence of fracture in the base cohort, the sensitivity analyses showed that studying first fractures only did not influence the study findings. Additional research investigating the validity of fracture algorithms would benefit studies.

## CONCLUSIONS

The results of this study provide evidence of the potential for opioid use to increase the risk of the user sustaining fractures, particularly during the initial weeks of starting and restarting opioids. These findings complement the results from existing studies that used between-participant study designs and demonstrate the utility of self-controlled methods for pharmacoepidemiologic research.

## Supplementary Material

Web_Material_kwab042
