# Supplementary material for: Opioids and the Risk of Fracture: A Self-Controlled Case Series Study in the Clinical Practice Research Datalink
Source: Am J Epidemiol. 2021 Feb 19;190(7):1324–31. doi: 10.1093/aje/kwab042 (PMC8245882; doi:10.1093/aje/kwab042)
Supplement: Web_Material_kwab042 [file web_material_kwab042.pdf]

Opioids and the Risk of Fracture: a Self-Controlled Case Series Study in the Clinical Practice Research Datalink

Emily J. Peach, Fiona A. Pearce, Jack Gibson, Andrew J. Cooper, Li-Chia Chen and Roger D. Knaggs

Web Materials Table of Contents

**Web Table 1**	Fracture codes
**Web Table 2**	Included opioid drugs and equianalgesic ratios
**Web Figure 1**	Overview of opioid prescription preparation process
**Web Figure 2**	Proximity of fracture events to opioid initiation and definition of the pre-exposure risk period
**Web Figure 3**	Curtailment of overlapping risk periods
**Web Table 3**	Fracture risk increasing drugs (excluding opioids)
**Web Figure 4**	Selection of study cohort
**Web Table 4**	Sensitivity analyses
**Web Figure 5**	Risk of fracture when exposed to opioids by anatomical site
**Web Figure 6**	Comparison of aIRRs in primary analysis and after excluding fractures to spine, chest, low back and pelvis
**Web Figure 7**	Risk of falls when exposed to opioids

Web Table 1. Fracture codes

code	system	description
S2...11	Read	Arm fracture
S28..11	Read	Ill-defined fracture of arm
S28z.00	Read	Ill-defined fractures of upper limb NOS
S28..00	Read	Ill-defined fractures of upper limb
S280.00	Read	Closed ill-defined fractures of upper limb
S281.00	Read	Open ill-defined fractures of upper limb
S29..11	Read	Multiple fractures of arm
S294000	Read	Cl fractures involving multiple regions of both upper limbs
SR12000	Read	Closed fractures involving multiple regions of one upp limb
S120900	Read	Closed fracture multiple ribs
S120100	Read	Closed fracture of one rib
S120000	Read	Closed fracture of rib, unspecified
S112.00	Read	Closed fracture of thoracic spine with spinal cord lesion
S120.00	Read	Closed fracture rib
S122.00	Read	Closed fracture sternum
S102.00	Read	Closed fracture thoracic vertebra
S102z00	Read	Closed fracture thoracic vertebra not otherwise specified
S102000	Read	Closed fracture thoracic vertebra, burst
S102500	Read	Closed fracture thoracic vertebra, posterior arch
S102300	Read	Closed fracture thoracic vertebra, spinous process
S102200	Read	Closed fracture thoracic vertebra, spondylolysis
S102400	Read	Closed fracture thoracic vertebra, transverse process
S102100	Read	Closed fracture thoracic vertebra, wedge
S150000	Read	Closed multiple fractures of thoracic spine
N331011	Read	Collapse of thoracic vertebra
N331F00	Read	Collapse of thoracic vertebra
N331K00	Read	Collapse of thoracic vertebra due to osteoporosis
S127.00	Read	Fracture of rib
S128.00	Read	Fracture of sternum
S15..00	Read	Fracture of thoracic vertebra
S150.00	Read	Multiple fractures of thoracic spine
S29..12	Read	Multiple rib fractures
S102y00	Read	Other specified closed fracture thoracic vertebra
S12z.11	Read	Rib fracture NOS
N331000	Read	Pathological fracture of thoracic vertebra
S103.00	Read	Open fracture thoracic vertebra
S103100	Read	Open fracture thoracic vertebra, wedge
S103500	Read	Open fracture thoracic vertebra, posterior arch
S120200	Read	Closed fracture of two ribs
S120300	Read	Closed fracture of three ribs
S120400	Read	Closed fracture of four ribs
S120500	Read	Closed fracture of five ribs
S120600	Read	Closed fracture of six ribs

S120700	Read	Closed fracture of seven ribs
S120800	Read	Closed fracture of eight or more ribs
S120A00	Read	Cough fracture
S120z00	Read	Closed fracture of rib(s) NOS
S121.00	Read	Open fracture rib
S121000	Read	Open fracture of rib, unspecified
S121200	Read	Open fracture of two ribs
S121700	Read	Open fracture of seven ribs
S121900	Read	Open fracture multiple ribs
S121z00	Read	Open fracture of rib(s) NOS
S123.00	Read	Open fracture sternum
S127000	Read	Multiple fractures of ribs
S127100	Read	Cough fracture of ribs
S12z.12	Read	Sternum fracture NOS
S150100	Read	Open multiple fracture of thoracic spine
S29..13	Read	Multiple fractures of sternum
S4J0000	Read	Closed fracture-dislocation of sternum
S4J1000	Read	Open fracture-dislocation of sternum
S4J1200	Read	Open fracture-dislocation sterno-clavicular joint, anterior
S4J2000	Read	Closed fracture-subluxation of sternum
S4J3000	Read	Open fracture-subluxation of sternum
S12X000	Read	Closed fracture of bony thorax part unspecified
S12y000	Read	Closed fracture of other parts of bony thorax
S352300	Read	Closed fracture cuboid
S352700	Read	Closed fracture metatarsal
S350.00	Read	Closed fracture of calcaneus
S360.00	Read	Closed fracture of one or more phalanges of foot
7K1LB00	Read	Closed reduction of fracture of hallux
7K1LA00	Read	Closed reduction of fracture of toe
S356.00	Read	Fracture of metatarsal bone
S36..00	Read	Fracture of one or more phalanges of foot
S363.00	Read	Fracture of other toe
S355.00	Read	Fracture of talus
S35..11	Read	Metatarsal bone fracture
S3x4.00	Read	Multiple fractures of foot
S362100	Read	Open fracture of great toe
S350.12	Read	Os calcis fracture
S36..11	Read	Toe fracture
7K1L900	Read	Closed reduction of fracture of metatarsus
S35..12	Read	Tarsal bone fracture
S350.11	Read	Heel bone fracture
S350000	Read	Closed fracture calcaneus, extra-articular
S350100	Read	Closed fracture calcaneus, intra-articular
S351.00	Read	Open fracture of calcaneus
S351100	Read	Open fractures calcaneus, intra-articular
S352.00	Read	Closed fracture of other tarsal and metatarsal bones

S352.11	Read	March fracture
S352000	Read	Closed fracture of tarsal bone, unspecified
S352100	Read	Closed fracture of talus
S352111	Read	Closed fracture of astragalus
S352200	Read	Closed fracture navicular
S352400	Read	Closed fracture medial cuneiform
S352500	Read	Closed fracture intermediate cuneiform
S352600	Read	Closed fracture lateral cuneiform
S352800	Read	Closed fracture talus, head
S352900	Read	Closed fracture talus, neck
S352A00	Read	Closed fracture talus, body
S352B00	Read	Closed fracture metatarsal base
S352C00	Read	Closed fracture metatarsal shaft
S352D00	Read	Closed fracture metatarsal neck
S352E00	Read	Closed fracture metatarsal head
S352F00	Read	Closed fracture metatarsal, multiple
S352G00	Read	Closed tarsal fractures, multiple
S352H00	Read	Closed fracture of cuneiforms
S352J00	Read	Closed fracture of base of fifth metatarsal
S352z00	Read	Closed fracture of one or more tarsal + metatarsal bones NOS
S353.00	Read	Open fracture of other tarsal and metatarsal bones
S353000	Read	Open fracture of tarsal bone, unspecified
S353100	Read	Open fracture of talus
S353200	Read	Open fracture navicular
S353300	Read	Open fracture cuboid
S353400	Read	Open fracture medial cuneiform
S353500	Read	Open fracture intermediate cuneiform
S353600	Read	Open fracture lateral cuneiform
S353700	Read	Open fracture metatarsal
S353800	Read	Open fracture talus, head
S353900	Read	Open fracture talus, neck
S353A00	Read	Open fracture talus, body
S353B00	Read	Open fracture metatarsal base
S353C00	Read	Open fracture metatarsal shaft
S353D00	Read	Open fracture metatarsal neck
S353E00	Read	Open fracture metatarsal head
S353F00	Read	Open fracture metatarsal, multiple
S353H00	Read	Open fracture cuneiforms
S353J00	Read	Open fracture of base of fifth metatarsal
S353z00	Read	Open fracture of tarsal and metatarsal bones NOS
S360000	Read	Closed fracture proximal phalanx, toe
S360100	Read	Closed fracture middle phalanx, toe
S360200	Read	Closed fracture distal phalanx, toe
S360300	Read	Closed fracture multiple phalanges, toe
S361.00	Read	Open fracture of one or more phalanges of foot
S361000	Read	Open fracture proximal phalanx, toe

S361100	Read	Open fracture middle phalanx, toe
S361200	Read	Open fracture distal phalanx, toe
S361300	Read	Open fracture multiple phalanges, toe
S362000	Read	Closed fracture of great toe
S4H0.00	Read	Closed fracture-dislocation foot
S4H0000	Read	Closed fracture-dislocation, subtalar joint
S4H0100	Read	Closed fracture-dislocation, midtarsal joint
S4H0200	Read	Closed fracture-dislocation, tarsometatarsal joint
S4H0400	Read	Closed fracture-dislocation, IPJ, single toe
S4H0600	Read	Closed fracture-dislocation, IPJ, multiple toes
S4H1.00	Read	Open fracture-dislocation, foot
S4H1000	Read	Open fracture-dislocation, subtalar joint
S4H1100	Read	Open fracture-dislocation, midtarsal joint
S4H1200	Read	Open fracture-dislocation, tarsometatarsal joint
S4H1300	Read	Open fracture-dislocation, metatarsophalangeal joint, single
S4H1400	Read	Open fracture-dislocation, IPJ, single toe
S4H1600	Read	Open fracture-dislocation, IPJ, multiple toes
S4H2.00	Read	Closed fracture-subluxation, foot
S4H2000	Read	Closed fracture-subluxation, subtalar joint
S4H2100	Read	Closed fracture-subluxation, midtarsal joint
S4H2200	Read	Closed fracture-subluxation, tarsometatarsal joint
S4H2400	Read	Closed fracture-subluxation, IPJ, single toe
S4H2600	Read	Closed fracture-subluxation, IPJ, multiple toes
S4H3.00	Read	Open fracture-subluxation, foot
S4H3300	Read	Open fracture-subluxation, metatarsophalangeal joint, single
S4H3400	Read	Open fracture-subluxation, IPJ, single toe
Syu5400	Read	[X]Fracture of forearm, unspecified
Syu5300	Read	[X]Fracture of other parts of forearm
S234D00	Read	Closed fracture distal radius, extra-articular, other type
S234C00	Read	Closed fracture distal radius, intra-articular, die-punch
S234E00	Read	Closed fracture distal radius, intra-articular, other type
S234500	Read	Closed fracture distal ulna, unspecified
S234z00	Read	Closed fracture of forearm, lower end, NOS
S234000	Read	Closed fracture of forearm, lower end, unspecified
S23x000	Read	Closed fracture of forearm, unspecified
S230.00	Read	Closed fracture of proximal radius and ulna
S230400	Read	Closed fracture of proximal ulna, comminuted
S23x100	Read	Closed fracture of radius (alone), unspecified
S234.00	Read	Closed fracture of radius and ulna, lower end
S23xz00	Read	Closed fracture of radius and ulna, NOS
S232.00	Read	Closed fracture of radius and ulna, shaft
S232z00	Read	Closed fracture of radius and ulna, shaft, NOS
S23x.00	Read	Closed fracture of radius and ulna, unspecified part
S232000	Read	Closed fracture of radius, shaft, unspecified
S234200	Read	Closed fracture of the distal radius, unspecified
S230500	Read	Closed fracture of the proximal ulna

S232100	Read	Closed fracture of the radial shaft
S23x300	Read	Closed fracture of the radius and ulna
S232200	Read	Closed fracture of the ulnar shaft
S23x200	Read	Closed fracture of ulna (alone), unspecified
S230100	Read	Closed fracture olecranon, extra-articular
S234600	Read	Closed fracture radius and ulna, distal
S232300	Read	Closed fracture radius and ulna, middle
S230A00	Read	Closed fracture radius and ulna, proximal
S230600	Read	Closed fracture radius, head
S230700	Read	Closed fracture radius, neck
S4C0000	Read	Closed fracture-dislocation distal radio-ulnar joint
S234800	Read	Closed Galeazzi fracture
7K1LE00	Read	Closed reduction of fracture of elbow
S23C.00	Read	Fracture of lower end of both ulna and radius
S23B.00	Read	Fracture of lower end of radius
S23..00	Read	Fracture of radius and ulna
S23z.00	Read	Fracture of radius and ulna, NOS
S23x111	Read	Fracture of radius NOS
S239.00	Read	Fracture of shaft of radius
S238.00	Read	Fracture of shaft of ulna
S23A.00	Read	Fracture of shafts of both ulna and radius
S23x211	Read	Fracture of ulna NOS
S237.00	Read	Fracture of upper end of radius
7K1LL00	Read	Closed reduction of fracture of radius and or ulna
S23..11	Read	Forearm fracture
S230000	Read	Closed fracture of proximal forearm, unspecified part
S230200	Read	Closed fracture of ulna, coronoid
S230300	Read	Closed Monteggia's fracture
S230800	Read	Closed fracture proximal radius, comminuted
S230900	Read	Closed fracture of the proximal radius
S230B00	Read	Closed fracture olecranon, intra-articular
S230z00	Read	Closed fracture of proximal forearm not otherwise specified
S231.00	Read	Open fracture of proximal radius and ulna
S231000	Read	Open fracture of proximal forearm, unspecified
S231100	Read	Open fracture olecranon, extra-articular
S231200	Read	Open fracture of ulna, coronoid
S231300	Read	Open Monteggia's fracture
S231500	Read	Open fracture of the proximal ulna
S231600	Read	Open fracture radial head
S231700	Read	Open fracture radial neck
S231800	Read	Open fracture proximal radius, comminuted
S231900	Read	Open fracture of the proximal radius
S231A00	Read	Open fracture radius and ulna, proximal
S231B00	Read	Open fracture olecranon, intra-articular
S231z00	Read	Open fracture of forearm, upper end, NOS
S233.00	Read	Open fracture of radius and ulna, shaft

S233000	Read	Open fracture of radius, shaft, unspecified
S233100	Read	Open fracture of the radial shaft
S233200	Read	Open fracture of the ulnar shaft
S233300	Read	Open fracture radius and ulna, middle
S233z00	Read	Open fracture of radius and ulna, shaft, NOS
S234211	Read	Dupuytren's fracture, radius - closed
S234300	Read	Closed fracture of ulna, styloid process
S234400	Read	Closed fracture of ulna, lower epiphysis
S234G00	Read	Greenstick fracture of distal radius
S235.00	Read	Open fracture of radius and ulna, lower end
S235000	Read	Open fracture of forearm, lower end, unspecified
S235200	Read	Open fracture of the distal radius, unspecified
S235211	Read	Dupuytren's fracture, radius - open
S235300	Read	Open fracture of ulna, styloid process
S235400	Read	Open fracture of ulna, lower epiphysis
S235500	Read	Open fracture distal ulna - other
S235600	Read	Open fracture radius and ulna, distal
S235800	Read	Open Galeazzi fracture
S235C00	Read	Open fracture distal radius, intra-articular, die-punch
S235D00	Read	Open fracture distal radius, extra-articular other type
S235E00	Read	Open fracture distal radius, intra-articular other type
S235z00	Read	Open fracture of forearm, lower end, NOS
S23y.00	Read	Open fracture of radius and ulna, unspecified part
S23y000	Read	Open fracture of forearm, unspecified
S23y100	Read	Open fracture of radius (alone), unspecified
S23y200	Read	Open fracture of ulna (alone), unspecified
S23y300	Read	Open fracture of the radius and ulna
S23yz00	Read	Open fracture of radius and ulna, NOS
S293.00	Read	Multiple fractures of forearm
S024000	Read	Closed fracture maxilla
S020.00	Read	Closed fracture nose
S024100	Read	Closed fracture zygoma
7K1LD00	Read	Closed reduction of fracture of nasal bone
S02x000	Read	Fracture of alveolus, closed
S01..00	Read	Fracture of base of skull
S01z.00	Read	Fracture of base of skull NOS
S02..00	Read	Fracture of face bones
S022.12	Read	Fracture of lower jaw, closed
S024.00	Read	Fracture of malar or maxillary bones, closed
S028300	Read	Fracture of mandible
S022.00	Read	Fracture of mandible, closed
S022z00	Read	Fracture of mandible, closed, NOS
S028000	Read	Fracture of nasal bones
S02x100	Read	Fracture of orbit NOS, closed
S028100	Read	Fracture of orbital floor
S0...00	Read	Fracture of skull

S02z.11	Read	Jaw fracture NOS
S02B.00	Read	Le Fort II fracture maxilla
S04..12	Read	Multiple skull fractures
S01..15	Read	Occiput bone fracture
S021.00	Read	Open fracture nose
7J03100	Read	Reduction of fracture of nasal bones NEC other
7J03200	Read	Reduction of fracture of zygomatic bones
S03z.00	Read	Skull fracture NOS
7206100	Read	Open reduction of fracture of orbit
7206200	Read	Removal of fixation from fracture of orbit
7206400	Read	Open reduction of fracture of orbit and internal fixation
7206700	Read	Packing of maxilla to correct blow-out fracture of orbit
7206800	Read	Internal fixation of fracture of orbit
7403600	Read	Outfracture of turbinates of nose
7403900	Read	Surgical outfracture of turbinate of nose
7J02200	Read	Elevation of depressed fracture of cranium
7J02300	Read	Repair of fracture of cranium NEC
7J03.00	Read	Reduction of fracture of facial bone
7J03000	Read	Reduction of fracture of nasoethmoid complex of bones
7J03300	Read	Reduction of closed fracture of orbit bone
7J03y00	Read	Other specified reduction of fracture of facial bone
7J03z00	Read	Reduction of fracture of facial bone NOS
7J12.00	Read	Reduction of fracture of mandible
7J12.11	Read	Reduction of fracture of jaw NEC
7J12000	Read	Reduction of fracture of alveolus of mandible
7J12100	Read	Open reduction of fracture of mandible NEC
7J12200	Read	Closed reduction of fracture of mandible NEC
7J12y00	Read	Other specified reduction of fracture of mandible
7J12z00	Read	Reduction of fracture of mandible NOS
7J13.00	Read	Reduction of fracture of maxilla
7J13000	Read	Reduction of fracture of alveolus of maxilla
7J13100	Read	Open reduction of fracture of maxilla NEC
7J13200	Read	Closed reduction of fracture of maxilla NEC
7J13300	Read	Reduction of blowout fracture of orbital floor
7J13400	Read	Reduction of Le Fort 1 fracture of maxilla
7J13500	Read	Reduction of Le Fort 2 fracture of maxilla
7J13600	Read	Reduction of Le Fort 3 fracture of maxilla
7J13y00	Read	Other specified reduction of fracture of maxilla
7J13z00	Read	Reduction of fracture of maxilla NOS
7J17700	Read	Traction for fracture of jaw
S000.00	Read	Closed fracture vault of skull without intracranial injury
S001.00	Read	Closed fracture vault of skull with intracranial injury
S002.00	Read	Open fracture vault of skull without intracranial injury
S003.00	Read	Open fracture vault of skull with intracranial injury
S01..11	Read	Anterior fossa fracture
S01..12	Read	Ethmoid sinus fracture

S01..13	Read	Frontal sinus fracture
S01..14	Read	Middle fossa fracture
S01..16	Read	Orbital roof fracture
S01..17	Read	Posterior fossa fracture
S01..18	Read	Sphenoid bone fracture
S01..19	Read	Temporal bone fracture
S010.00	Read	Closed fracture base of skull without intracranial injury
S011.00	Read	Closed fracture base of skull with intracranial injury
S012.00	Read	Open fracture base skull without mention intracranial injury
S013.00	Read	Open fracture base of skull with intracranial injury
S020.11	Read	Closed fracture nasal bone
S021.11	Read	Open fracture nasal bone
S022000	Read	Closed fracture mandible (site unspecified)
S022100	Read	Closed fracture of mandible, condylar process
S022200	Read	Closed fracture of mandible, subcondylar
S022300	Read	Closed fracture of mandible, coronoid process
S022400	Read	Closed fracture of mandible, ramus, unspecified
S022500	Read	Closed fracture of mandible, angle of jaw
S022600	Read	Closed fracture of mandible, symphysis of body
S022700	Read	Closed fracture of mandible, alveolar border of body
S022800	Read	Closed fracture of mandible, body, other and unspecified
S022x00	Read	Closed fracture of mandible, multiple sites
S023000	Read	Open fracture mandible (site unspecified)
S023100	Read	Open fracture of mandible, condylar process
S023200	Read	Open fracture of mandible, subcondylar
S023400	Read	Open fracture of mandible, ramus, unspecified
S023500	Read	Open fracture of mandible, angle of jaw
S023600	Read	Open fracture of mandible, symphysis of body
S023700	Read	Open fracture of mandible, alveolar border of body
S023800	Read	Open fracture of mandible, body, other and unspecified
S023x00	Read	Open fracture of mandible, multiple sites
S025000	Read	Open fracture maxilla
S025100	Read	Open fracture zygoma
S026.00	Read	Closed orbital blow-out fracture
S027.00	Read	Open orbital blow-out fracture
S02A.00	Read	Le Fort I fracture maxilla
S02C.00	Read	Le Fort III fracture maxilla
S02x.00	Read	Closed fracture other facial bone
S02y.00	Read	Open fracture other facial bone
S03..00	Read	Other and unqualified skull fractures
S030.00	Read	Closed fracture of skull NOS without intracranial injury
S031.00	Read	Closed fracture of skull NOS with intracranial injury
S033.00	Read	Open fracture of skull NOS with intracranial injury
S03z.11	Read	Depressed skull fracture NOS
S04..00	Read	Multiple fractures involving skull or face with other bones
S04..11	Read	Multiple face fractures

S044.00	Read	Multiple fractures involving skull and facial bones
S04z.00	Read	Multiple fractures involving skull/face with other bones NOS
7K1J011	Read	Cl red intracaps frac neck femur fix-Garden cannulated screw
7K1J012	Read	Cl red intracaps fract neck femur fix - Smith-Petersen nail
S300400	Read	Closed fracture head of femur
S302011	Read	Closed fracture of femur, greater trochanter
S302400	Read	Closed fracture of femur, intertrochanteric
S302012	Read	Closed fracture of femur, lesser trochanter
S300y11	Read	Closed fracture of femur, subcapital
S300A00	Read	Closed fracture of femur, upper epiphysis
S30y.00	Read	Closed fracture of neck of femur NOS
S302.00	Read	Closed fracture of proximal femur, pertrochanteric
S30w.00	Read	Closed fracture of unspecified proximal femur
S300300	Read	Closed fracture proximal femur, basicervical
S302100	Read	Closed fracture proximal femur, intertrochanteric, two part
S300200	Read	Closed fracture proximal femur, midcervical section
S300y00	Read	Closed fracture proximal femur, other transcervical
S300600	Read	Closed fracture proximal femur, subcapital, Garden grade I
S300700	Read	Closed fracture proximal femur, subcapital, Garden grade II
S300800	Read	Closed fracture proximal femur, subcapital, Garden grade III
S300900	Read	Closed fracture proximal femur, subcapital, Garden grade IV
S300.00	Read	Closed fracture proximal femur, transcervical
S300z00	Read	Closed fracture proximal femur, transcervical, NOS
S300100	Read	Closed fracture proximal femur, transepiphyseal
S300311	Read	Closed fracture, base of neck of femur
7K1L400	Read	Closed reduction of fracture of hip
7K1Jd00	Read	Closed reduction of intracapsular # NOF internal fixat DHS
S302z00	Read	Cls # of proximal femur, pertrochanteric section, NOS
S300000	Read	Cls # prox femur, intracapsular section, unspecified
S300500	Read	Cls # prox femur, subcapital, Garden grade unspec.
S302300	Read	Cls # proximal femur, intertrochanteric, comminuted
S302000	Read	Cls # proximal femur, trochanteric section, unspecified
7K1J013	Read	Cls red+int fxn prox femoral #+Richard's cannulat hip screw
7K1J000	Read	Cls red+int fxn proximal femoral #+screw/nail device alone
7K1D01E	Read	DHS - Dynamic hip screw primary fixation of neck of femur
7K1D01F	Read	Dynamic hip screw primary fixation of neck of femur
S30..00	Read	Fracture of neck of femur
S30..11	Read	Hip fracture
S30y.11	Read	Hip fracture NOS
S303000	Read	Open # of proximal femur, trochanteric section, unspecified
S301311	Read	Open fracture base of neck of femur
S303400	Read	Open fracture of femur, intertrochanteric
S301y11	Read	Open fracture of femur, subcapital
S301A00	Read	Open fracture of femur, upper epiphysis
S30z.00	Read	Open fracture of neck of femur NOS
S303.00	Read	Open fracture of proximal femur, pertrochanteric

S303z00	Read	Open fracture of proximal femur, pertrochanteric, NOS
S30x.00	Read	Open fracture of unspecified proximal femur
S303300	Read	Open fracture proximal femur, intertrochanteric, comminuted
S303100	Read	Open fracture proximal femur, intertrochanteric, two part
S301y00	Read	Open fracture proximal femur, other transcervical
S301.00	Read	Open fracture proximal femur, transcervical
S301100	Read	Open fracture proximal femur, transepiphyseal
S301600	Read	Open fracture proximal femur,subcapital, Garden grade I
S301700	Read	Open fracture proximal femur,subcapital, Garden grade II
S301800	Read	Open fracture proximal femur,subcapital, Garden grade III
S301900	Read	Open fracture proximal femur,subcapital, Garden grade IV
S301500	Read	Open fracture proximal femur,subcapital, Garden grade unspec
S301000	Read	Opn # proximal femur, intracapsular section, unspecified
S304.00	Read	Pertrochanteric fracture
7K1JC00	Read	Prim cls rd+int fxn prox fem #+screw/nail+intramdulry device
7K1D013	Read	Prim op red # nck femur & op fix - Deyerle multiple hip pin
7K1D012	Read	Prim op red # nck femur & op fix- Charnley compression screw
7K1D01D	Read	Prim op red # nck femur & op fix- Zickel intramed nail plate
7K1DE00	Read	Prim op red frac neck fem op fix us prox fem nail antirotatn
7K1D017	Read	Prim open red # neck femur & op fix - McLaughlin nail plate
7K1D011	Read	Prim open reduct # neck femur & op fix - Blount nail plate
7K1D014	Read	Prim open reduct # neck femur & op fix - Holt nail
7K1D015	Read	Prim open reduct # neck femur & op fix - Jewett nail plate
7K1D018	Read	Prim open reduct # neck femur & op fix - Neufield nail plate
7K1D019	Read	Prim open reduct # neck femur & op fix - Pugh nail plate
7K1D01A	Read	Prim open reduct # neck femur & op fix - Richards screw
7K1D01B	Read	Prim open reduct # neck femur & op fix - Ross Brown nail
7K1JB00	Read	Primary cls red+int fxn prox fem #+screw/nail device alone
7K1JD00	Read	Primary cls red+int fxn prox fem #+screw/nail+plate device
7K1K500	Read	Primary cls reduction+external fixation proximal femoral #
7K1K300	Read	Primary external fixation(without reduction) prox femoral #
7K1J500	Read	Primary int fxn(no red) prox fem #+screw/nail device alone
7K1J700	Read	Primary int fxn(no red) prox fem #+screw/nail+plate device
7K1J600	Read	Primary int fxn(no red) prox fem #+scrw/nail+intramed device
7K1G200	Read	Primary open reduction+external fixation of femoral fracture
7K1D700	Read	Prmy open red+int fxn prox fem #+screw/nail+intramed device
7K1D600	Read	Prmy open red+int fxn prox femoral #+screw/nail device alone
7K1D000	Read	Prmy open red+int fxn prox femoral #+screw/nail+plate device
S130000	Read	Closed fracture acetabulum, anterior lip alone
S130100	Read	Closed fracture acetabulum, posterior lip alone
S130200	Read	Closed fracture acetabulum, anterior column
S130300	Read	Closed fracture acetabulum, posterior column
S130400	Read	Closed fracture acetabulum, floor
S130600	Read	Closed fracture acetabulum, double column unspecified
S130y00	Read	Other specified closed fracture acetabulum
S130z00	Read	Closed fracture acetabulum NOS

S131.00	Read	Open fracture acetabulum
S131y00	Read	Other specified open fracture acetabulum
S131z00	Read	Open fracture acetabulum NOS
S301400	Read	Open fracture head, femur
S303011	Read	Open fracture of femur, greater trochanter
S4E0.00	Read	Closed fracture-dislocation, hip joint
S4E1.00	Read	Open fracture-dislocation, hip joint
S4E2.00	Read	Closed fracture-subluxation, hip joint
S3...00	Read	Fracture of lower limb
S3...11	Read	Leg fracture
7K1LC00	Read	Closed reduction of fracture of lower limb
S370.00	Read	Closed fracture of lower limb, level unspecified
S371.00	Read	Open fracture of lower limb, level unspecified
S3x..00	Read	Other, multiple and ill-defined fractures of lower limb
S3x0.00	Read	Other, multiple and ill-defined closed fractures lower limb
S3x1.00	Read	Other, multiple and ill-defined open fractures of lower limb
SR15000	Read	Cl fractures involving multiple regions upper with lower lmb
SyuL400	Read	[X]Sequelae of other fractures of lower limb
S130.00	Read	Closed fracture acetabulum
S104.00	Read	Closed fracture lumbar vertebra
S104000	Read	Closed fracture lumbar vertebra, burst
S104500	Read	Closed fracture lumbar vertebra, posterior arch
S104300	Read	Closed fracture lumbar vertebra, spinous process
S104200	Read	Closed fracture lumbar vertebra, spondylolysis
S104400	Read	Closed fracture lumbar vertebra, transverse process
S104600	Read	Closed fracture lumbar vertebra, tricolumnar
S104100	Read	Closed fracture lumbar vertebra, wedge
S114.00	Read	Closed fracture of lumbar spine with spinal cord lesion
S13y.00	Read	Closed fracture of pelvis NOS
S134600	Read	Closed fracture pelvis, iliac wing
S134100	Read	Closed fracture pelvis, ischium
S132100	Read	Closed fracture pelvis, multiple pubic rami - stable
S132000	Read	Closed fracture pelvis, single pubic ramus
S132.00	Read	Closed fracture pubis
S132z00	Read	Closed fracture pubis NOS
S4J0100	Read	Closed fracture-dislocation of pelvis
S4J2100	Read	Closed fracture-subluxation of pelvis
N331111	Read	Collapse of lumbar vertebra
N331G00	Read	Collapse of lumbar vertebra
N331J00	Read	Collapse of lumbar vertebra due to osteoporosis
S10B400	Read	Fracture of acetabulum
S10B200	Read	Fracture of coccyx
S10B.00	Read	Fracture of lumbar spine and pelvis
S10B000	Read	Fracture of lumbar vertebra
S10B500	Read	Fracture of pubis
S13..00	Read	Fracture or disruption of pelvis

SR11.00	Read	Fractures involving thorax with lower back and pelvis
S10B600	Read	Multiple fractures of lumbar spine and pelvis
S13z.00	Read	Open fracture of pelvis NOS
S134.00	Read	Other or multiple closed fracture of pelvis
S134z00	Read	Other or multiple closed fracture of pelvis NOS
N331100	Read	Pathological fracture of lumbar vertebra
S105.00	Read	Open fracture lumbar vertebra
S105000	Read	Open fracture lumbar vertebra, burst
S105100	Read	Open fracture lumbar vertebra, wedge
S105400	Read	Open fracture lumbar vertebra, transverse process
S106.00	Read	Closed fracture sacrum
S106000	Read	Closed compression fracture sacrum
S106100	Read	Closed vertical fracture of sacrum
S107.00	Read	Open fracture sacrum
S107000	Read	Open compression fracture sacrum
S107100	Read	Open vertical fracture of sacrum
S108.00	Read	Closed fracture pelvis, coccyx
S109.00	Read	Open fracture pelvis, coccyx
S115.00	Read	Open fracture of lumbar spine with spinal cord lesion
S116.00	Read	Closed fracture of sacrum with spinal cord lesion
S116z00	Read	Closed fracture of sacrum with spinal cord lesion NOS
S117.00	Read	Open fracture of sacrum with spinal cord lesion
S117300	Read	Open fracture of sacrum with other spinal cord injury
S118.00	Read	Closed fracture of coccyx with spinal cord lesion
S118z00	Read	Closed fracture of coccyx with spinal cord lesion NOS
S132200	Read	Closed fracture pelvis, multiple pubic rami - unstable
S132y00	Read	Other specified closed fracture pubis
S133.00	Read	Open fracture of pubis
S133000	Read	Open fracture pelvis, single pubic ramus
S133100	Read	Open fracture pelvis, multiple pubic rami - stable
S133200	Read	Open fracture pelvis, multiple pubic rami - unstable
S133y00	Read	Other specified open fracture of pubis
S133z00	Read	Open fracture of pubis NOS
S134000	Read	Closed fracture of ilium, unspecified
S134300	Read	Closed fracture pelvis, ischial tuberosity
S134400	Read	Closed fracture pelvis, anterior superior iliac spine
S134500	Read	Closed fracture pelvis, anterior inferior iliac spine
S134700	Read	Closed vertical fracture of ilium
S134800	Read	Closed fracture dislocation of sacro-iliac joint
S135.00	Read	Other or multiple open fracture of pelvis
S135000	Read	Open fracture of ilium, unspecified
S135100	Read	Open fracture pelvis, ischium
S135300	Read	Open fracture pelvis, ischial tuberosity
S135400	Read	Open fracture pelvis, anterior superior iliac spine
S135600	Read	Open fracture pelvis, iliac wing
S135800	Read	Open fracture dislocation of sacro-iliac joint

S135y00	Read	Other open fracture of pelvis
S135z00	Read	Other/multiple open fracture of pelvis NOS
S4J1100	Read	Open fracture-dislocation of pelvis
S4J3100	Read	Open fracture-subluxation of pelvis
S344.00	Read	Closed fracture ankle, bimalleolar
S342.00	Read	Closed fracture ankle, lateral malleolus
S34x.00	Read	Closed fracture ankle, unspecified
S334.00	Read	Closed fracture distal tibia
S334000	Read	Closed fracture distal tibia, extra-articular
S339000	Read	Closed fracture of distal fibula
S33x100	Read	Closed fracture of fibula, unspecified part, NOS
S330.00	Read	Closed fracture of tibia and fibula, proximal
S33x200	Read	Closed fracture of tibia and fibula, unspecified part
S33x000	Read	Closed fracture of tibia, unspecified part, NOS
S332.00	Read	Closed fracture of tibia/fibula, shaft
S320400	Read	Closed fracture patella, comminuted (stellate)
S330100	Read	Closed fracture proximal fibula
S330300	Read	Closed fracture proximal tibia, medial condyle (plateau)
S332100	Read	Closed fracture shaft of fibula
S4F0.00	Read	Closed fracture-dislocation, knee joint
S4F2.00	Read	Closed fracture-subluxation, knee joint
7K1L800	Read	Closed reduction of fracture of ankle
7K1L600	Read	Closed reduction of fracture of knee
7K1L700	Read	Closed reduction of fracture of tibia and or fibula
S34..00	Read	Fracture of ankle
S34z.00	Read	Fracture of ankle, NOS
S339.00	Read	Fracture of fibula alone
S349.00	Read	Fracture of lateral malleolus
S338.00	Read	Fracture of lower end of tibia
S35..00	Read	Fracture of one or more tarsal and metatarsal bones
S32..00	Read	Fracture of patella
S32z.00	Read	Fracture of patella, NOS
S337.00	Read	Fracture of shaft of tibia
S33..00	Read	Fracture of tibia and fibula
S336.00	Read	Fracture of upper end of tibia
S4F..00	Read	Fracture-dislocation or subluxation knee
S3x3.00	Read	Multiple fractures of lower leg
S345.00	Read	Open fracture ankle, bimalleolar
S339100	Read	Open fracture of distal fibula
S33yz00	Read	Open fracture of tibia and fibula, unspecified part, NOS
S33y000	Read	Open fracture of tibia, unspecified part, NOS
S3xz.00	Read	Other, multiple and ill-defined fractures of lower limb NOS
S344.12	Read	Pott's fracture - ankle
7K1F500	Read	Primary open reduction fracture patella fixat tension band
S320.00	Read	Closed fracture of the patella
S320000	Read	Closed fracture patella, transverse

S320100	Read	Closed fracture patella, proximal pole
S320200	Read	Closed fracture patella, distal pole
S320300	Read	Closed fracture patella, vertical
S321.00	Read	Open fracture of the patella
S321000	Read	Open fracture patella, transverse
S321100	Read	Open fracture patella, proximal pole
S321200	Read	Open fracture patella, distal pole
S321400	Read	Open fracture patella, comminuted (stellate)
S330000	Read	Closed fracture of the proximal tibia
S330011	Read	Closed fracture of tibial condyles
S330012	Read	Closed fracture of tibial tuberosity
S330200	Read	Closed fracture of tibia and fibula, proximal
S330400	Read	Closed fracture proximal tibia, lateral condyle (plateau)
S330500	Read	Closed fracture proximal tibia, bicondylar
S330600	Read	Closed fracture spine, tibia
S330700	Read	Closed fracture tubercle, tibia
S330800	Read	Closed fracture fibula, head
S330900	Read	Closed fracture fibula, neck
S330z00	Read	Closed fracture of tibia and fibula, proximal NOS
S331.00	Read	Open fracture of tibia and fibula, proximal
S331000	Read	Open fracture of the proximal tibia
S331011	Read	Open fracture of tibial condyles
S331012	Read	Open fracture of tibial tuberosity
S331100	Read	Open fracture proximal fibula
S331200	Read	Open fracture of tibia and fibula, proximal
S331300	Read	Open fracture proximal tibia, medial condyle (plateau)
S331400	Read	Open fracture proximal tibia, lateral condyle (plateau)
S331600	Read	Open fracture spine, tibia
S331700	Read	Open fracture tubercle, tibia
S331800	Read	Open fracture fibula, head
S331900	Read	Open fracture fibula, neck
S331A00	Read	Open fracture tibial plateau
S331z00	Read	Open fracture of tibia and fibula, proximal NOS
S332000	Read	Closed fracture shaft of tibia
S332200	Read	Closed fracture of tibia and fibula, shaft
S332z00	Read	Closed fracture of tibia and fibula, shaft, NOS
S333.00	Read	Open fracture of tibia/fibula, shaft
S333000	Read	Open fracture shaft of tibia
S333100	Read	Open fracture shaft of fibula
S333200	Read	Open fracture of tibia and fibula, shaft
S333z00	Read	Open fracture of tibia and fibula, shaft, NOS
S334100	Read	Closed fracture distal tibia, intra-articular
S335.00	Read	Open fracture distal tibia
S335000	Read	Open fracture distal tibia, extra-articular
S335100	Read	Open fracture distal tibia, intra-articular
S33B.00	Read	Open fracture of distal tibia and fibula

S33C.00	Read	Closed fracture of distal tibia and fibula
S33x.00	Read	Closed fracture of tibia and fibula, unspecified part, NOS
S33x.11	Read	Lower leg fracture NOS
S33xz00	Read	Closed fracture of tibia and fibula, unspecified part, NOS
S33y.00	Read	Open fracture of tibia and fibula, unspecified part, NOS
S33y100	Read	Open fracture of fibula, unspecified part, NOS
S33y200	Read	Open fracture of tibia and fibula, unspecified part
S340.00	Read	Closed fracture ankle, medial malleolus
S341.00	Read	Open fracture ankle, medial malleolus
S342000	Read	Closed fracture ankle, lateral malleolus, low
S342100	Read	Closed fracture ankle, lateral malleolus, high
S343.00	Read	Open fracture ankle, lateral malleolus
S343000	Read	Open fracture ankle, lateral malleolus, low
S343100	Read	Open fracture ankle, lateral malleolus, high
S344.11	Read	Dupuytren's fracture, fibula
S344000	Read	Closed fracture ankle, bimalleolar, low fibular fracture
S344100	Read	Closed fracture ankle, bimalleolar, high fibular fracture
S345000	Read	Open fracture ankle, bimalleolar, low fibular fracture
S345100	Read	Open fracture ankle, bimalleolar, high fibular fracture
S346.00	Read	Closed fracture ankle, trimalleolar
S346000	Read	Closed fracture ankle, trimalleolar, low fibular fracture
S346100	Read	Closed fracture ankle, trimalleolar, high fibular fracture
S347.00	Read	Open fracture ankle, trimalleolar
S347000	Read	Open fracture ankle, trimalleolar, low fibular fracture
S347100	Read	Open fracture ankle, trimalleolar, high fibular fracture
S34y.00	Read	Open fracture ankle, unspecified
S4F1.00	Read	Open fracture-dislocation, knee joint
S4F3.00	Read	Open fracture-subluxation, knee joint
S4F4.00	Read	Closed fracture-dislocation, patello-femoral joint
S4F5.00	Read	Open fracture-dislocation, patello-femoral joint
S4F6.00	Read	Closed fracture-subluxation, patello-femoral joint
S4F7.00	Read	Open fracture-subluxation, patello-femoral joint
S4G0.00	Read	Closed fracture-dislocation, ankle joint
S4G1.00	Read	Open fracture-dislocation, ankle joint
S4G2.00	Read	Closed fracture-subluxation, ankle joint
S4G3.00	Read	Open fracture-subluxation, ankle joint
SC0X.00	Read	Sequelae of other fracture of thorax and pelvis
SR10000	Read	Closed fractures involving head with neck
SR16000	Read	Closed fracture inv thorax wth low back and pelvis and limbs
SR1z.00	Read	Multiple fractures, unspecified
SR1z000	Read	[X]Closed multiple fractures unspecified
SR1z100	Read	[X]Open multiple fractures unspecified
S100.00	Read	Closed fracture of cervical spine
S110.00	Read	Closed fracture of cervical spine with cord lesion
N331E00	Read	Collapse of cervical vertebra
N331H00	Read	Collapse of cervical vertebra due to osteoporosis

S10A000	Read	Fracture of first cervical vertebra
S10A.00	Read	Fracture of neck
S10A100	Read	Fracture of second cervical vertebra
S10A200	Read	Multiple fractures of cervical spine
N331A00	Read	Osteoporosis + pathological fracture cervical vertebrae
N331C00	Read	Pathological fracture of cervical vertebra
S100000	Read	Closed fracture of unspecified cervical vertebra
S100100	Read	Closed fracture atlas
S100111	Read	C1 vertebra closed fracture - no spinal cord lesion
S100200	Read	Closed fracture axis
S100211	Read	C2 vertebra closed fracture without spinal cord lesion
S100300	Read	Closed fracture of third cervical vertebra
S100311	Read	C3 vertebra closed fracture without spinal cord lesion
S100400	Read	Closed fracture of fourth cervical vertebra
S100411	Read	C4 vertebra closed fracture without spinal cord lesion
S100500	Read	Closed fracture of fifth cervical vertebra
S100511	Read	C5 vertebra closed fracture without spinal cord lesion
S100600	Read	Closed fracture of sixth cervical vertebra
S100611	Read	C6 vertebra closed fracture without spinal cord lesion
S100700	Read	Closed fracture of seventh cervical vertebra
S100711	Read	C7 vertebra closed fracture without spinal cord lesion
S100800	Read	Closed fracture atlas, isolated arch or articular process
S100900	Read	Closed fracture atlas, comminuted
S100A00	Read	Closed fracture axis, odontoid process
S100B00	Read	Closed fracture axis, spondylolysis
S100C00	Read	Closed fracture axis, spinous process
S100D00	Read	Closed fracture axis, transverse process
S100E00	Read	Closed fracture axis, posterior arch
S100G00	Read	Closed fracture cervical vertebra, burst
S100H00	Read	Closed fracture cervical vertebra, wedge
S100J00	Read	Closed fracture cervical vertebra, spondylolysis
S100K00	Read	Closed fracture cervical vertebra, spinous process
S100L00	Read	Closed fracture cervical vertebra, transverse process
S100M00	Read	Closed fracture cervical vertebra, posterior arch
S100x00	Read	Multiple closed fractures of cervical vertebrae
S100z00	Read	Closed fracture of cervical spine not otherwise specified
S101.00	Read	Open fracture of cervical spine
S101000	Read	Open fracture of unspecified cervical vertebra
S101100	Read	Open fracture atlas
S101111	Read	C1 vertebra open fracture without spinal cord lesion
S101200	Read	Open fracture axis
S101211	Read	C2 vertebra open fracture without spinal cord lesion
S101311	Read	C3 vertebra open fracture without spinal cord lesion
S101500	Read	Open fracture of fifth cervical vertebra
S101511	Read	C5 vertebra open fracture without spinal cord lesion
S101600	Read	Open fracture of sixth cervical vertebra

S101611	Read	C6 vertebra open fracture without spinal cord lesion
S101711	Read	C7 vertebra open fracture without spinal cord lesion
S101900	Read	Open fracture atlas, comminuted
S101A00	Read	Open fracture axis, odontoid process
S101x00	Read	Multiple open fractures of cervical vertebrae
S125100	Read	Closed fracture of hyoid bone
S126100	Read	Open fracture of hyoid bone
Syu4300	Read	[X]Fracture of other parts of shoulder and upper arm
Syu4400	Read	[X]Fracture of shoulder and upper arm, unspecified
S200300	Read	Closed fracture clavicle, lateral end
S224100	Read	Closed fracture distal humerus, supracondylar
S200.00	Read	Closed fracture of clavicle
S224500	Read	Closed fracture of distal humerus, trochlea
S224000	Read	Closed fracture of elbow, unspecified part
S222000	Read	Closed fracture of humerus NOS
S222100	Read	Closed fracture of humerus, shaft
S222.00	Read	Closed fracture of humerus, shaft or unspecified part
S222z00	Read	Closed fracture of humerus, shaft or unspecified part NOS
S220500	Read	Closed fracture of humerus, upper epiphysis
S220z00	Read	Closed fracture of proximal humerus not otherwise specified
S220200	Read	Closed fracture of proximal humerus, anatomical neck
S220000	Read	Closed fracture of proximal humerus, unspecified part
S224.00	Read	Closed fracture of the distal humerus
S220.00	Read	Closed fracture of the proximal humerus
S220700	Read	Closed fracture proximal humerus, four part
S220300	Read	Closed fracture proximal humerus, greater tuberosity
S220400	Read	Closed fracture proximal humerus, head
S220100	Read	Closed fracture proximal humerus, neck
S220600	Read	Closed fracture proximal humerus, three part
S210100	Read	Closed fracture scapula, acromion
S210300	Read	Closed fracture scapula, glenoid
S4A0.00	Read	Closed fracture-dislocation shoulder
S4C2000	Read	Closed fracture-subluxation, distal radio-ulnar jt
7K1LG00	Read	Closed reduction of fracture of shoulder
S20..11	Read	Collar bone fracture
S224.11	Read	Elbow fracture - closed
S20..00	Read	Fracture of clavicle
S22..00	Read	Fracture of humerus
S22z.00	Read	Fracture of humerus NOS
S228.00	Read	Fracture of lower end of humerus
S21..00	Read	Fracture of scapula
S227.00	Read	Fracture of shaft of humerus
S226.00	Read	Fracture of upper end of humerus
S2...00	Read	Fracture of upper limb
S4A..00	Read	Fracture-dislocation or subluxation shoulder
S292.00	Read	Multiple fractures of clavicle, scapula and humerus

7K1LF00	Read	Closed reduction of fracture of humerus
7K1LN00	Read	Closed reduction of fracture of upper limb
S200000	Read	Closed fracture of clavicle, unspecified part
S200100	Read	Closed fracture clavicle, medial end
S200200	Read	Closed fracture clavicle, shaft
S200z00	Read	Closed fracture of clavicle NOS
S201.00	Read	Open fracture of clavicle
S201000	Read	Open fracture of clavicle, unspecified part
S201100	Read	Open fracture clavicle, medial end
S201200	Read	Open fracture clavicle, shaft
S201300	Read	Open fracture clavicle, lateral end
S201z00	Read	Open fracture of clavicle NOS
S21..11	Read	Shoulder blade fracture
S210.00	Read	Closed fracture of scapula
S210000	Read	Closed fracture of scapula, unspecified part
S210200	Read	Closed fracture scapula, coracoid
S210400	Read	Closed fracture scapula, blade
S210500	Read	Closed fracture scapula, spine
S210600	Read	Closed fracture scapula, neck
S210z00	Read	Closed fracture of scapula NOS
S211.00	Read	Open fracture of scapula
S211000	Read	Open fracture of scapula, unspecified part
S211100	Read	Open fracture scapula, acromion
S211200	Read	Open fracture scapula, coracoid
S211300	Read	Open fracture scapula, glenoid
S211400	Read	Open fracture scapula, blade
S211600	Read	Open fracture scapula, neck
S211z00	Read	Open fracture of scapula NOS
S221.00	Read	Open fracture of the proximal humerus
S221.11	Read	Shoulder fracture - open
S221000	Read	Open fracture of proximal humerus, unspecified part
S221100	Read	Open fracture proximal humerus, neck
S221200	Read	Open fracture of proximal humerus, anatomical neck
S221300	Read	Open fracture proximal humerus, greater tuberosity
S221400	Read	Open fracture proximal humerus, head
S221500	Read	Open fracture of humerus, upper epiphysis
S221600	Read	Open fracture proximal humerus, three part
S221700	Read	Open fracture proximal humerus, four part
S221z00	Read	Open fracture of proximal humerus not otherwise specified
S223.00	Read	Open fracture of humerus, shaft or unspecified part
S223000	Read	Open fracture of humerus NOS
S223100	Read	Open fracture of humerus, shaft
S223z00	Read	Open fracture of humerus, shaft or unspecified part NOS
S224200	Read	Closed fracture distal humerus, lateral condyle
S224300	Read	Closed fracture distal humerus, medial condyle
S224400	Read	Closed fracture of distal humerus, condyle(s) unspecified

S224600	Read	Closed fracture distal humerus, lateral epicondyle
S224700	Read	Closed fracture distal humerus, medial epicondyle
S224800	Read	Closed fracture distal humerus, capitellum
S224900	Read	Closed fracture distal humerus, bicondylar (T-Y fracture)
S224x00	Read	Closed fracture of distal humerus, multiple
S224z00	Read	Closed fracture of distal humerus, not otherwise specified
S225.00	Read	Open fracture of the distal humerus
S225.11	Read	Elbow fracture - open
S225000	Read	Open fracture of elbow, unspecified part
S225100	Read	Open fracture distal humerus, supracondylar
S225200	Read	Open fracture distal humerus, lateral condyle
S225300	Read	Open fracture distal humerus, medial condyle
S225400	Read	Open fracture of distal humerus, condyle(s) unspecified
S225500	Read	Open fracture of distal humerus, trochlea
S225600	Read	Open fracture distal humerus, lateral epicondyle
S225700	Read	Open fracture distal humerus, medial epicondyle
S225800	Read	Open fracture distal humerus, capitellum
S225900	Read	Open fracture distal humerus, bicondylar (T-Y fracture)
S225x00	Read	Open fracture of distal humerus, multiple
S225z00	Read	Open fracture of distal humerus, not otherwise specified
S292000	Read	Closed multiple fractures of clavicle, scapula and humerus
S292100	Read	Open multiple fractures of clavicle, scapula and humerus
S4A0000	Read	Closed fracture-dislocation shoulder joint
S4A0100	Read	Closed fracture-dislocation acromio-clavicular joint
S4A1.00	Read	Open fracture-dislocation shoulder
S4A1000	Read	Open fracture-dislocation shoulder joint
S4A1100	Read	Open fracture-dislocation acromio-clavicular joint
S4A2.00	Read	Closed fracture-subluxation shoulder
S4A2000	Read	Closed fracture-subluxation shoulder joint
S4A2100	Read	Closed fracture-subluxation acromio-clavicular joint
S4A3.00	Read	Open fracture-subluxation shoulder
S4A3100	Read	Open fracture-subluxation acromio-clavicular joint
S4B0.00	Read	Closed fracture-dislocation elbow
S4B0000	Read	Closed fracture-dislocation elbow joint
S4B0100	Read	Closed fracture-dislocation superior radio-ulnar joint
S4B1.00	Read	Open fracture-dislocation elbow
S4B1000	Read	Open fracture-dislocation elbow joint
S4B1100	Read	Open fracture-dislocation superior radio-ulnar joint
S4B2.00	Read	Closed fracture-subluxation elbow
S4B2000	Read	Closed fracture-subluxation elbow joint
S4B2100	Read	Closed fracture-subluxation superior radio-ulnar joint
S4B3.00	Read	Open fracture-subluxation elbow
Syu4200	Read	[X]Multiple fractures of clavicle, scapula and humerus
Nyu6700	Read	[X]Collapsed vertebra in diseases classified elsewhere
7J41500	Read	Balloon kyphoplasty of fracture of spine
S11x.00	Read	Closed fracture of spine with spinal cord lesion unspecified

S10x.00	Read	Closed fracture of spine, unspecified,
S112z00	Read	Closed fracture of thoracic spine with cord lesion NOS
S114100	Read	Closed spinal fracture with complete lumbar cord lesion
S114000	Read	Closed spinal fracture with unspecified lumbar cord lesion
S112700	Read	Cls spinal fracture with complete thorac cord lesion, T7-12
S112A00	Read	Cls spinal fracture with posterior thorac cord lesion, T7-12
S112600	Read	Cls spinal fracture with unspec thoracic cord lesion, T7-12
S112000	Read	Cls spinal fracture with unspec thoracic cord lesion,T1-6
S112100	Read	Cls spinal fracture wth complete thoracic cord lesion,T1-6
N331.11	Read	Collapse of spine NOS
N331L00	Read	Collapse of vertebra due to osteoporosis NOS
N331.12	Read	Collapse of vertebra NOS
N331D00	Read	Collapsed vertebra NOS
7J41.00	Read	Decompression of fracture of spine
N1y1.00	Read	Fatigue fracture of vertebra
7J43100	Read	Fixation of fracture of spine using Harrington rod
S11..00	Read	Fracture of spine with spinal cord lesion
S11z.00	Read	Fracture of spine with spinal cord lesion NOS
S10..00	Read	Fracture of spine without mention of spinal cord injury
S10z.00	Read	Fracture of spine without mention of spinal cord lesion NOS
S10..11	Read	Fracture of transverse process spine - no spinal cord lesion
S11..12	Read	Fracture of vertebra with spinal cord lesion
S10..12	Read	Fracture of vertebra without spinal cord lesion vert
14G8.00	Read	H/O: vertebral fracture
7J42400	Read	Halo skull traction for fracture of spine
N331800	Read	Osteoporosis + pathological fracture lumbar vertebrae
N331900	Read	Osteoporosis + pathological fracture thoracic vertebrae
N331.14	Read	Osteoporotic vertebral collapse
7J41000	Read	Complex decompression of fracture of spine
7J41100	Read	Anterior decompression of fracture of spine
7J41200	Read	Posterior decompression of fracture of spine
7J41300	Read	Vertebroplasty of fracture of spine
7J41400	Read	Posterior decompression of fracture of spine NEC
7J41y00	Read	Other specified decompression of fracture of spine
7J41z00	Read	Decompression of fracture of spine NOS
7J42.00	Read	Other reduction of fracture of spine
7J42.11	Read	Other reduction of fracture of spine and stabilisation
7J42000	Read	Open reduction of fracture of spine & excis facet of spine
7J42100	Read	Open reduction of fracture of spine NEC
7J42200	Read	Manipulative reduction of fracture of spine
7J42300	Read	Spinal extension traction for fracture of spine
7J42500	Read	Spinal traction for fracture of spine NEC
7J42600	Read	Primary bedrest stabilisation of spinal fracture
7J42700	Read	Primary collar stabilisation of spinal fracture
7J42900	Read	Primary cast stabilisation of spinal fracture
7J42B00	Read	Primary other external stabilisation of spinal fracture

7J42C00	Read	Revision to bedrest stabilisation of spinal fracture
7J42D00	Read	Revision to collar stabilisation of spinal fracture
7J42G00	Read	Revision to external fixation stabilisation spinal fracture
7J42J00	Read	Primary closed reduction spinal fracture alone
7J42L00	Read	Primary cls reduction spinal fracture+bedrest stabilisation
7J42M00	Read	Primary cls reduc spinal fracture+skull traction stabilisatn
7J42y00	Read	Other specified other reduction of fracture of spine
7J42z00	Read	Other reduction of fracture of spine NOS
7J43.00	Read	Fixation of fracture of spine
7J43.11	Read	Internal fixation of fracture of spine
7J43000	Read	Primary open reduc spinal fracture+internal fix+plate
7J43200	Read	Fixation of fracture of spine and skull traction HFQ
7J43211	Read	Barr skull traction for fracture of spine
7J43300	Read	Primary open reduc spinal fracture+internal fix+wire
7J43400	Read	Primary open reduc spinal fracture+internal fix+rod system
7J43700	Read	Primary open reduc spinal fracture+other internal fix
7J43900	Read	Rvsn open reduc spinal fracture+internal fix+plate
7J43A00	Read	Rvsn open reduc spinal fracture+internal fix+rod system
7J43C00	Read	Rvsn open reduc spinal fracture+internal fix+internl fixator
7J43E00	Read	Removal of fracture fixation device from spine
7J43y00	Read	Other specified fixation of fracture of spine
7J43z00	Read	Fixation of fracture of spine NOS
S10y.00	Read	Open fracture of spine, unspecified,
S110000	Read	Cls spinal fracture with unspec cervical cord lesion, C1-4
S110100	Read	Cls spinal fracture with complete cervcl cord lesion, C1-4
S110600	Read	Cls spinal fracture with unspec cervical cord lesion, C5-7
S110700	Read	Cls spinal fracture with complete cervcl cord lesion, C5-7
S110800	Read	Cls spinal fracture with anterior cervcl cord lesion, C5-7
S110z00	Read	Closed fracture of cervical spine with cord lesion NOS
S111.00	Read	Open fracture of cervical spine with spinal cord lesion
S113.00	Read	Open fracture of thoracic spine with spinal cord lesion
S113000	Read	Opn spinal fracture with unspec thoracic cord lesion, T1-6
S113A00	Read	Opn spinal fracture with posterior thorac cord lesion, T7-12
S114500	Read	Closed spinal fracture with cauda equina lesion
S312500	Read	Closed fracture distal femur, lateral condyle
S312300	Read	Closed fracture distal femur, supracondylar
S312000	Read	Closed fracture of distal femur, unspecified
S312100	Read	Closed fracture of femoral condyle, unspecified
S312.11	Read	Closed fracture of femur, distal end
S312200	Read	Closed fracture of femur, lower epiphysis
S310.00	Read	Closed fracture of femur, shaft or unspecified part
S310000	Read	Closed fracture of femur, unspecified part
S302200	Read	Closed fracture proximal femur, subtrochanteric
7K1L500	Read	Closed reduction of fracture of femur
S31z.00	Read	Fracture of femur, NOS
S315.00	Read	Fracture of lower end of femur

S314.00	Read	Fracture of shaft of femur
S3x2.00	Read	Multiple fractures of femur
S311.00	Read	Open fracture of femur, shaft or unspecified part
S31..00	Read	Other fracture of femur
S305.00	Read	Subtrochanteric fracture
S310011	Read	Thigh fracture NOS
S303200	Read	Open fracture proximal femur, subtrochanteric
S310012	Read	Upper leg fracture NOS
S310100	Read	Closed fracture shaft of femur
S310z00	Read	Closed fracture of shaft or unspecified part, NOS
S311000	Read	Open fracture of femur, unspecified part
S311100	Read	Open fracture shaft of femur
S311z00	Read	Open fracture of femur, shaft or unspecified part, NOS
S312.00	Read	Closed fracture distal femur
S312400	Read	Closed fracture distal femur, medial condyle
S312600	Read	Closed fracture distal femur, bicondylar (T-Y fracture)
S312x00	Read	Closed fracture distal femur, comminuted/intra-articular
S312z00	Read	Closed fracture of distal femur not otherwise specified
S313.00	Read	Open fracture distal femur
S313.11	Read	Open fracture of femur, distal end
S313000	Read	Open fracture distal femur, unspecified
S313100	Read	Open fracture of femoral condyle, unspecified
S313200	Read	Open fracture of femur, lower epiphysis
S313300	Read	Open fracture distal femur, supracondylar
S313400	Read	Open fracture distal femur, medial condyle
S313500	Read	Open fracture distal femur, lateral condyle
S313x00	Read	Open fracture distal femur, comminuted/intra-articular
S313z00	Read	Open fracture of distal femur not otherwise specified
SC3D400	Read	Sequelae of fracture of femur
S1...00	Read	Fracture of neck and trunk
NyuB000	Read	[X]Other osteoporosis with pathological fracture
NyuB800	Read	[X]Unspecified osteoporosis with pathological fracture
S3z0.00	Read	Closed fracture of bones, unspecified
N331500	Read	Drug-induced osteoporosis with pathological fracture
S3z..11	Read	Fracture NOS
N331700	Read	Fracture of bone in neoplastic disease
S3zz.00	Read	Fracture of bones NOS
S3z..00	Read	Fracture of unspecified bones
TC7..00	Read	Fracture, cause unspecified
N331N00	Read	Fragility fracture
N331M00	Read	Fragility fracture due to unspecified osteoporosis
S3z0000	Read	Greenstick fracture
N331600	Read	Idiopathic osteoporosis with pathological fracture
7K1L100	Read	Manipulation of fracture of bone NEC
N331N11	Read	Minimal trauma fracture
N331M11	Read	Minimal trauma fracture due to unspecified osteoporosis

7K1L.00	Read	Other closed reduction of fracture of bone
N331.00	Read	Pathological fracture
N331B00	Read	Postmenopausal osteoporosis with pathological fracture
7K1D100	Read	Prim open reduct fract long bone & fixation rigid
7K1D400	Read	Prim open reduction fragment of bone & fixation using
7K1LV00	Read	Primary closed reduction of fracture alone
7K1D.00	Read	Primary open reduction fracture bone & intramedull fixation
7K1JH00	Read	Primary wire fixation of fracture
7K1D800	Read	Prmy open reduction #+locked reamed intramedullary nail fxtn
7K1E000	Read	Prmy open reduction of #+internal fixation with plate NEC
7K1E800	Read	Prmy open reduction of #+internal fixation with screw(s)
7K1D511	Read	K wiring of fracture
7K1Dy00	Read	Prim open reduction fracture bone & intramedullary fixatn OS
7K1Dz00	Read	Prim open reduction fracture bone & intramedull fixation NOS
7K1Ez00	Read	Prim open reduction fracture bone & extramedull fixation NOS
7K1F.00	Read	Primary open reduction of intraarticular fracture of bone
7K1F300	Read	Primary intraarticular fixation intraartic fracture bone NEC
7K1F400	Read	Prim extraarticular reduction intraartic fracture bone NEC
7K1Fy00	Read	Primary open reduction of intraarticular fracture bone OS
7K1Fz00	Read	Primary open reduction of intraarticular fracture bone NOS
7K1G.00	Read	Other primary open reduction of fracture of bone
7K1G000	Read	Prmy open reduction of fracture and skeletal traction
7K1G100	Read	Prmy open reduction of fracture and external fixation
7K1G300	Read	Primary open reduction of fracture alone
7K1G400	Read	Primary open reduction of fracture and cast immobilisation
7K1G500	Read	Primary open reduction of fracture and functional bracing
7K1G600	Read	Primary open reduction of fracture and skin traction
7K1Gy11	Read	Primary open reduction of bone fracture & external fixation
7K1Gz00	Read	Other primary open reduction of fracture of bone NOS
7K1H.00	Read	Secondary open reduction of fracture of bone
7K1H.11	Read	Revision to open reduction of fracture of bone
7K1H200	Read	Secondary open reduction of intraarticular fracture of bone
7K1H400	Read	Secondary open reduct fracture bone & external fixation HFQ
7K1H900	Read	Revision to open reduction of fracture alone
7K1HD00	Read	Revision to open reduction of fracture and skeletal traction
7K1HE00	Read	Revision to open reduction of fracture and external fixation
7K1Hy00	Read	Other specified secondary open reduction of fracture of bone
7K1Hz00	Read	Secondary open reduction of fracture of bone NOS
7K1J.00	Read	Closed (or no) reduction of fracture and internal fixation
7K1J300	Read	Closed reduction fracture small bone & fixation using screw
7K1JJ00	Read	Revision to wire fixation of fracture
7K1JK00	Read	Primary closed reduction of fracture and wire fixation
7K1JL00	Read	Revision to closed reduction of fracture and wire fixation
7K1Jy00	Read	Closed reduction of bone fracture and internal fixation OS
7K1Jz00	Read	Closed reduction of bone fracture and internal fixation NOS
7K1K.00	Read	Closed (or no) reduction of fracture and external fixation

7K1K000	Read	Closed reduction fracture bone and fixation to skeleton HFQ
7K1K200	Read	Remanipulation of fracture of bone and external fixation HFQ
7K1K700	Read	Primary functional bracing of fracture
7K1K800	Read	Primary external fixation of fracture
7K1K900	Read	Other primary external immobilisation of fracture
7K1KA00	Read	Revision to functional bracing of fracture
7K1KB00	Read	Revision to external fixation of fracture
7K1KC00	Read	Other revision to external immobilisation of fracture
7K1KE00	Read	Primary closed reduction of fracture and external fixation
7K1Ky00	Read	Closed reduction of bone fracture and external fixation OS
7K1Kz00	Read	Closed reduction of bone fracture and external fixation NOS
7K1L011	Read	Manipulation of fracture and skeletal traction NEC
7K1L211	Read	Remanipulation of fracture and skeletal traction NEC
7K1L300	Read	Remanipulation of fracture of bone NEC
7K1LT00	Read	Primary closed reduction of fracture and cast immobilisation
7K1LW0 0	Read	Primary closed reduction of fracture and skin traction
7K1LX00	Read	Revision to closed reduction of fracture alone
7K1LZ00	Read	Primary skin traction of fracture
7K1La00	Read	Revision to skin traction of fracture
7K1Lb00	Read	Primary cast immobilisation of fracture
7K1Lc00	Read	Revision to cast immobilisation of fracture
7K1Ld00	Read	Primary arthroscopic reduction of fracture
7K1Le00	Read	Primary arthroscopic reduction and fixation of fracture
7K1Lf00	Read	Revision to arthroscopic reduction of fracture
7K1Lg00	Read	Revision to arthroscopic reduction and fixation of fracture
7K1Ly00	Read	Other specified other closed reduction of fracture of bone
7K1Lz00	Read	Other closed reduction of fracture of bone NOS
7K1N900	Read	Primary skeletal traction of fracture
7K1T100	Read	Debridement of open fracture
7K1Y.00	Read	Second closed reduction fracture bone and internal fixation
7K1Y100	Read	Remanip fracture long bone and rigid internal fixation NEC
7K1Yy00	Read	OS second closed reduct fracture bone and internal fixation
7K6F200	Read	Primary open reduction of fracture dislocation of joint NEC
7K6FE00	Read	Primary open reduction of fracture dislocation alone
7K6GN0 0	Read	Closed reduction fracture disloc joint & internal fixation
7K6GX00	Read	Primary closed reduction of fracture dislocation alone
7K6H200	Read	Secondary open reduction fracture dislocation of joint NEC
7K6H400	Read	Revision to closed reduction of fracture dislocation alone
7K6H411	Read	Remanipulation of fracture dislocation alone
7K6H700	Read	Secondary open reduction fracture disloc joint & fixation
7K6HX00	Read	Revision to open reduction fracture dislocation alone
7K6Hh00	Read	Sec open red fracture dislocat joint and intern fixation NEC
82...11	Read	Closed reduction of fracture
N1y2.00	Read	Pars interarticularis stress fracture
N331.13	Read	Sponanteous fracture
N331200	Read	Postoophorectomy osteoporosis with pathological fracture

N331300	Read	Osteoporosis of disuse with pathological fracture
N331400	Read	Postsurgical malabsorption osteoporosis with path fracture
N331y00	Read	Other specified pathological fracture
N331z00	Read	Pathological fracture NOS
N338.00	Read	Malunion and nonunion of fracture
N338000	Read	Malunion of fracture
N338100	Read	Pseudoarthrosis - fracture nonunion
N338111	Read	Nonunion of fracture
N338200	Read	Hypertrophic non-union of fracture
N338300	Read	Atrophic non-union of fracture
N338400	Read	Angular mal-union of fracture
N338500	Read	Rotational mal-union of fracture
N338600	Read	Delayed union of fracture
S00..11	Read	Frontal bone fracture
S00..12	Read	Parietal bone fracture
S140.00	Read	Closed fracture of ill-defined bone of trunk
S3z1.00	Read	Open fracture of bones, unspecified
S3z2.00	Read	Stress fracture
S4...13	Read	Fracture dislocations and fracture subluxations
S4J..00	Read	Other fracture-dislocation or subluxation
S4J0.00	Read	Other closed fracture-dislocation
S4J1.00	Read	Other open fracture-dislocation
S4J2.00	Read	Other closed fracture-subluxation
S4J3.00	Read	Other open fracture-subluxation
SC0z.11	Read	Delayed union of fracture
Zw02400	Read	[Q] Stress fracture
Zw02D00	Read	[Q] Open fracture grade 1
Zw02E00	Read	[Q] Open fracture grade 2
Syu6500	Read	[X]Fracture of other & unspecified parts of wrist and hand
S234A00	Read	Closd dorsal Barton's fracture
S234F00	Read	Closed Barton's fracture
S234100	Read	Closed Colles' fracture
S234A12	Read	Closed dorsal Barton fracture-subluxation
S234A11	Read	Closed dorsal Barton's fracture-dislocation
S240700	Read	Closed fracture capitate
S250600	Read	Closed fracture finger metacarpal
S250200	Read	Closed fracture finger metacarpal base
S250.00	Read	Closed fracture of metacarpal bone(s)
S260.00	Read	Closed fracture of one or more phalanges of hand
S240100	Read	Closed fracture of the scaphoid
S234B00	Read	Closed fracture radial styloid
S240500	Read	Closed fracture trapezium
S4C2.00	Read	Closed fracture-subluxation of the wrist
S4C2100	Read	Closed fracture-subluxation radiocarpal joint
7K1LH00	Read	Closed reduction of fracture of finger
S234700	Read	Closed Smith's fracture

S234912	Read	Closed volar Barton fracture-subluxation
S234900	Read	Closed volar Barton's fracture
S234911	Read	Closed volar Barton's fracture-dislocation
S26..11	Read	Finger fracture
S242.00	Read	Fracture at wrist and hand level
S2B..00	Read	Fracture of bone of hand
S24..00	Read	Fracture of carpal bone
S242100	Read	Fracture of first metacarpal bone
S25..00	Read	Fracture of metacarpal bone
S263.00	Read	Fracture of other finger
S242200	Read	Fracture of other metacarpal bone
S242000	Read	Fracture of scaphoid
S262.00	Read	Fracture of thumb
S4C..00	Read	Fracture-dislocation or subluxation of wrist
S4D..00	Read	Fracture-dislocation/subluxation finger/thumb
S25..11	Read	Hand fracture - metacarpal bone
S242300	Read	Multiple fractures of metacarpal bones
S235100	Read	Open Colles' fracture
S261000	Read	Open fracture of phalanx or phalanges, unspecified
S235B00	Read	Open fracture radial styloid
S234.11	Read	Wrist fracture - closed
7K1LJ00	Read	Closed reduction of fracture of thumb
7K1LK00	Read	Closed reduction of fracture of metacarpus
7K1LM00	Read	Closed reduction of fracture of wrist
S234111	Read	Smith's fracture - closed
S235.11	Read	Wrist fracture - open
S235111	Read	Smith's fracture - open
S235700	Read	Open Smith's fracture
S235900	Read	Open volar Barton's fracture
S235A00	Read	Open dorsal Barton's fracture
S235F00	Read	Open Barton's fracture
S24..11	Read	Hand fracture - carpal bone
S240.00	Read	Closed fracture of carpal bone
S240000	Read	Closed fracture of carpal bone, unspecified
S240200	Read	Closed fracture lunate
S240300	Read	Closed fracture triquetral
S240400	Read	Closed fracture pisiform
S240600	Read	Closed fracture trapezoid
S240800	Read	Closed fracture hamate
S240900	Read	Closed fracture hamate, hook
S240A00	Read	Closed fracture scaphoid, proximal pole
S240B00	Read	Closed fracture scaphoid, waist, transverse
S240C00	Read	Closed fracture scaphoid, waist, oblique
S240D00	Read	Closed fracture scaphoid, waist, comminuted
S240E00	Read	Closed fracture scaphoid, tuberosity
S240F00	Read	Closed fracture carpal bones, multiple

S240y00	Read	Closed fracture of other carpal bone
S240z00	Read	Closed fracture of carpal bone NOS
S241.00	Read	Open fracture of carpal bone
S241000	Read	Open fracture of carpal bone, unspecified
S241100	Read	Open fracture of the scaphoid
S241200	Read	Open fracture lunate
S241300	Read	Open fracture triquetral
S241400	Read	Open fracture pisiform
S241500	Read	Open fracture trapezium
S241600	Read	Open fracture trapezoid
S241700	Read	Open fracture capitate
S241800	Read	Open fracture hamate
S241900	Read	Open fracture hamate, hook
S241A00	Read	Open fracture scaphoid, proximal pole
S241B00	Read	Open fracture scaphoid, waist, transverse
S241C00	Read	Open fracture scaphoid, waist, oblique
S241D00	Read	Open fracture scaphoid, waist, comminuted
S241E00	Read	Open fracture scaphoid, tuberosity
S241z00	Read	Open fracture of carpal bone NOS
S250000	Read	Closed fracture of metacarpal bone (s), site unspecified
S250300	Read	Closed fracture finger metacarpal shaft
S250400	Read	Closed fracture finger metacarpal neck
S250500	Read	Closed fracture finger metacarpal head
S250700	Read	Closed fracture finger metacarpal, multiple
S250800	Read	Closed fracture of thumb metacarpal
S250A00	Read	Closed fracture thumb metacarpal shaft
S250B00	Read	Closed fracture thumb metacarpal neck
S250C00	Read	Closed fracture thumb metacarpal head
S250x00	Read	Closed fractures of multiple sites of unspecified metacarpus
S250z00	Read	Closed fracture of metacarpal bone(s) NOS
S251.00	Read	Open fracture of metacarpal bone(s)
S251000	Read	Open fracture of metacarpal bone(s), site unspecified
S251200	Read	Open fracture finger metacarpal base
S251300	Read	Open fracture finger metacarpal shaft
S251400	Read	Open fracture finger metacarpal neck
S251500	Read	Open fracture finger metacarpal head
S251600	Read	Open fracture finger metacarpal
S251700	Read	Open fracture finger metacarpal, multiple
S251800	Read	Open fracture of thumb metacarpal
S251A00	Read	Open fracture thumb metacarpal shaft
S251C00	Read	Open fracture thumb metacarpal head
S251x00	Read	Open fractures of multiple sites of unspecified metacarpus
S251z00	Read	Open fracture of metacarpal bone(s) NOS
S252.00	Read	Closed fracture sesamoid bone of hand
S253.00	Read	Open fracture sesamoid bone of hand
S26..12	Read	Thumb fracture excluding base

S260000	Read	Closed fracture of phalanx or phalanges, unspecified
S260300	Read	Closed fracture thumb proximal phalanx
S260400	Read	Closed fracture thumb proximal phalanx, base
S260500	Read	Closed fracture thumb proximal phalanx, shaft
S260600	Read	Closed fracture thumb proximal phalanx, neck
S260700	Read	Closed fracture thumb proximal phalanx, head
S260800	Read	Closed fracture thumb distal phalanx
S260900	Read	Closed fracture thumb distal phalanx, base
S260A00	Read	Closed fracture thumb distal phalanx, shaft
S260B00	Read	Closed fracture thumb distal phalanx, tuft
S260C00	Read	Closed fracture thumb distal phalanx, mallet
S260D00	Read	Closed fracture finger proximal phalanx
S260E00	Read	Closed fracture finger proximal phalanx, base
S260F00	Read	Closed fracture finger proximal phalanx, shaft
S260G00	Read	Closed fracture finger proximal phalanx, neck
S260H00	Read	Closed fracture finger proximal phalanx, head
S260J00	Read	Closed fracture finger proximal phalanx, multiple
S260K00	Read	Closed fracture finger middle phalanx
S260L00	Read	Closed fracture finger middle phalanx, base
S260M00	Read	Closed fracture finger middle phalanx, shaft
S260N00	Read	Closed fracture finger middle phalanx, neck
S260P00	Read	Closed fracture finger middle phalanx, head
S260Q00	Read	Closed fracture finger middle phalanx, multiple
S260R00	Read	Closed fracture finger distal phalanx
S260S00	Read	Closed fracture finger distal phalanx, base
S260T00	Read	Closed fracture finger distal phalanx, shaft
S260U00	Read	Closed fracture finger distal phalanx, tuft
S260V00	Read	Closed fracture finger distal phalanx, mallet
S260W0 0	Read	Closed fracture finger distal phalanx, multiple
S260x00	Read	Closed fractures of phalanx or phalanges, multiple sites
S260z00	Read	Closed fracture of one or more phalanges of hand NOS
S261.00	Read	Open fracture of one or more phalanges of hand
S261300	Read	Open fracture thumb proximal phalanx
S261400	Read	Open fracture thumb proximal phalanx, base
S261500	Read	Open fracture thumb proximal phalanx, shaft
S261600	Read	Open fracture thumb proximal phalanx, neck
S261700	Read	Open fracture thumb proximal phalanx, head
S261800	Read	Open fracture thumb distal phalanx
S261900	Read	Open fracture thumb distal phalanx, base
S261A00	Read	Open fracture thumb distal phalanx, shaft
S261B00	Read	Open fracture thumb distal phalanx, tuft
S261C00	Read	Open fracture thumb distal phalanx, mallet
S261D00	Read	Open fracture finger proximal phalanx
S261E00	Read	Open fracture finger proximal phalanx, base
S261F00	Read	Open fracture finger proximal phalanx, shaft
S261G00	Read	Open fracture finger proximal phalanx, neck

S261H00	Read	Open fracture finger proximal phalanx, head
S261J00	Read	Open fracture finger proximal phalanx, multiple
S261K00	Read	Open fracture finger middle phalanx
S261L00	Read	Open fracture finger middle phalanx, base
S261M00	Read	Open fracture finger middle phalanx, shaft
S261N00	Read	Open fracture finger middle phalanx, neck
S261P00	Read	Open fracture finger middle phalanx, head
S261R00	Read	Open fracture finger distal phalanx
S261S00	Read	Open fracture finger distal phalanx, base
S261T00	Read	Open fracture finger distal phalanx, shaft
S261U00	Read	Open fracture finger distal phalanx, tuft
S261V00	Read	Open fracture finger distal phalanx, mallet
S261W00	Read	Open fracture finger distal phalanx, multiple
S261x00	Read	Open fracture of phalanx or phalanges, multiple sites
S261z00	Read	Open fracture of one or more phalanges of hand NOS
S264.00	Read	Multiple fractures of fingers
S27..00	Read	Multiple fractures of hand bones
S270.00	Read	Closed multiple fractures of hand bones
S271.00	Read	Open multiple fractures of hand bones
S27z.00	Read	Multiple fractures of hand bones NOS
S4C0.00	Read	Closed fracture dislocation of wrist
S4C0100	Read	Closed fracture-dislocation radiocarpal joint
S4C0200	Read	Closed fracture-dislocation mid carpal
S4C0300	Read	Closed fracture-dislocation, carpometacarpal joint
S4C0400	Read	Closed fracture-dislocation lunate (volar)
S4C0500	Read	Closed fracture-dislocation peri-lunate (dorsal)
S4C0600	Read	Closed fracture-dislocation peri-lunate trans-scaphoid
S4C1.00	Read	Open fracture dislocation wrist
S4C1000	Read	Open fracture-dislocation, distal radio-ulnar joint
S4C1100	Read	Open fracture-dislocation radiocarpal joint
S4C1300	Read	Open fracture-dislocation carpometacarpal joint
S4C1600	Read	Open fracture-dislocation peri-lunate trans-scaphoid
S4C2200	Read	Closed fracture-subluxation mid carpal
S4C2300	Read	Closed fracture-subluxation, carpometacarpal joint
S4C2400	Read	Closed fracture-subluxation lunate (volar)
S4C2600	Read	Closed fracture-subluxation peri-lunate trans-scaphoid
S4C2y00	Read	Closed fracture-subluxation other carpal
S4C3.00	Read	Open fracture-subluxation of the wrist
S4C3000	Read	Open fracture-subluxation, distal radio-ulnar joint
S4C3100	Read	Open fracture-subluxation radiocarpal joint
S4C3300	Read	Open fracture-subluxation, carpometacarpal joint
S4C3600	Read	Open fracture-subluxation peri-lunate trans-scaphoid
S4D0.00	Read	Closed fracture-dislocation digit
S4D0000	Read	Closed fracture-dislocation digit, unspecified
S4D0100	Read	Closed fracture-dislocation, metacarpophalangeal joint
S4D0200	Read	Closed fracture-dislocation IPJ, unspecified

S4D0300	Read	Closed fracture-dislocation, distal interphalangeal joint
S4D0400	Read	Closed fracture-dislocation, proximal interphalangeal joint
S4D0500	Read	Closed fracture-dislocation, interphalangeal joint thumb
S4D0600	Read	Closed fracture-dislocation multiple digits
S4D1.00	Read	Open fracture-dislocation digit
S4D1000	Read	Open fracture-dislocation digit, unspecified
S4D1100	Read	Open fracture-dislocation, metacarpophalangeal joint
S4D1200	Read	Open fracture-dislocation IPJ, unspecified
S4D1300	Read	Open fracture-dislocation, distal interphalangeal joint
S4D1400	Read	Open fracture-dislocation, proximal interphalangeal joint
S4D1500	Read	Open fracture-dislocation, interphalangeal joint thumb
S4D1600	Read	Open fracture-dislocation multiple digits
S4D2.00	Read	Closed fracture-subluxation digit
S4D2000	Read	Closed fracture-subluxation digit, unspecified
S4D2100	Read	Closed fracture-subluxation, metacarpophalangeal joint
S4D2200	Read	Closed fracture-subluxation IPJ, unspecified
S4D2300	Read	Closed fracture-subluxation, distal interphalangeal joint
S4D2400	Read	Closed fracture-subluxation, proximal interphalangeal joint
S4D2500	Read	Closed fracture-subluxation, interphalangeal joint thumb
S4D2600	Read	Closed fracture-subluxation multiple digits
S4D3.00	Read	Open fracture-subluxation digit
S4D3100	Read	Open fracture-subluxation, metacarpophalangeal joint
S4D3300	Read	Open fracture-subluxation, distal interphalangeal joint
S4D3400	Read	Open fracture-subluxation, proximal interphalangeal joint
S4D3500	Read	Open fracture-subluxation, interphalangeal joint thumb
S4D3600	Read	Open fracture-subluxation multiple digits
SC3C000	Read	Sequelae of fracture at wrist and hand level
T02	ICD-10	Fractures involving multiple body regions
T02.0	ICD-10	Fractures involving head with neck
T02.1	ICD-10	Fractures involving thorax with lower back and pelvis
T02.2	ICD-10	Fractures involving multiple regions of one upper limb
T02.3	ICD-10	Fractures involving multiple regions of one lower limb
T02.4	ICD-10	Fractures involving multiple regions of both upper limbs
T02.5	ICD-10	Fractures involving multiple regions of both lower limbs
T02.6	ICD-10	Fractures involving multiple regions of upper limb(s) with lower limb(s)
T02.7	ICD-10	Fractures involving thorax with lower back and pelvis with limb(s)
T02.8	ICD-10	Fractures involving other combinations of body regions
T02.9	ICD-10	Multiple fractures, unspecified
T08	ICD-10	Fracture of spine, level unspecified
T10	ICD-10	Fracture of upper limb, level unspecified
T12	ICD-10	Fracture of lower limb, level unspecified
T14.2	ICD-10	Fracture of unspecified body region
S02	ICD-10	Fracture of skull and facial bones
S02.0	ICD-10	Fracture of vault of skull
S02.1	ICD-10	Fracture of base of skull
S02.2	ICD-10	Fracture of nasal bones

S02.3	ICD-10	Fracture of orbital floor
S02.4	ICD-10	Fracture of malar and maxillary bones
S02.5	ICD-10	Fracture of tooth
S02.6	ICD-10	Fracture of mandible
S02.7	ICD-10	Multiple fractures involving skull and facial bones
S02.8	ICD-10	Fractures of other skull and facial bones
S02.9	ICD-10	Fracture of skull and facial bones, part unspecified
S12	ICD-10	Fracture of neck
S12.0	ICD-10	Fracture of first cervical vertebra
S12.1	ICD-10	Fracture of second cervical vertebra
S12.2	ICD-10	Fracture of other specified cervical vertebra
S12.7	ICD-10	Multiple fractures of cervical spine
S12.8	ICD-10	Fracture of other parts of neck
S12.9	ICD-10	Fracture of neck, part unspecified
S22	ICD-10	Fracture of rib(s), sternum and thoracic spine
S22.0	ICD-10	Fracture of thoracic vertebra
S22.1	ICD-10	Multiple fractures of thoracic spine
S22.2	ICD-10	Fracture of sternum
S22.3	ICD-10	Fracture of rib
S22.4	ICD-10	Multiple fractures of ribs
S22.5	ICD-10	Flail chest
S22.8	ICD-10	Fracture of other parts of bony thorax
S22.9	ICD-10	Fracture of bony thorax, part unspecified
S32	ICD-10	Fracture of lumbar spine and pelvis
S32.0	ICD-10	Fracture of lumbar vertebra
S32.1	ICD-10	Fracture of sacrum
S32.2	ICD-10	Fracture of coccyx
S32.3	ICD-10	Fracture of ilium
S32.4	ICD-10	Fracture of acetabulum
S32.5	ICD-10	Fracture of pubis
S32.7	ICD-10	Multiple fractures of lumbar spine and pelvis
S32.8	ICD-10	Fracture of other and unspecified parts of lumbar spine and pelvis
S42	ICD-10	Fracture of shoulder and upper arm
S42.0	ICD-10	Fracture of clavicle
S42.1	ICD-10	Fracture of scapula
S42.2	ICD-10	Fracture of upper end of humerus
S42.3	ICD-10	Fracture of shaft of humerus
S42.4	ICD-10	Fracture of lower end of humerus
S42.7	ICD-10	Multiple fractures of clavicle, scapula and humerus
S42.8	ICD-10	Fracture of other parts of shoulder and upper arm
S42.9	ICD-10	Fracture of shoulder girdle, part unspecified
S52	ICD-10	Fracture of forearm
S52.0	ICD-10	Fracture of upper end of ulna
S52.1	ICD-10	Fracture of upper end of radius
S52.2	ICD-10	Fracture of shaft of ulna
S52.3	ICD-10	Fracture of shaft of radius

S52.4	ICD-10	Fracture of shafts of both ulna and radius
S52.5	ICD-10	Fracture of lower end of radius
S52.6	ICD-10	Fracture of lower end of both ulna and radius
S52.7	ICD-10	Multiple fractures of forearm
S52.8	ICD-10	Fracture of other parts of forearm
S52.9	ICD-10	Fracture of forearm, part unspecified
S62	ICD-10	Fracture of wrist and hand level
S62.0	ICD-10	Fracture of navicular [scaphoid] bone of hand
S62.1	ICD-10	Fracture of other carpal bone(s)
S62.2	ICD-10	Fracture of first metacarpal bone
S62.3	ICD-10	Fracture of other metacarpal bone
S62.4	ICD-10	Multiple fractures of metacarpal bones
S62.5	ICD-10	Fracture of thumb
S62.6	ICD-10	Fracture of other finger
S62.7	ICD-10	Multiple fractures of fingers
S62.8	ICD-10	Fracture of other and unspecified parts of wrist and hand
S72	ICD-10	Fracture of femur
S72.0	ICD-10	Fracture of neck of femur
S72.1	ICD-10	Pertrochanteric fracture
S72.2	ICD-10	Subtrochanteric fracture
S72.3	ICD-10	Fracture of shaft of femur
S72.4	ICD-10	Fracture of lower end of femur
S72.7	ICD-10	Multiple fractures of femur
S72.8	ICD-10	Fractures of other parts of femur
S72.9	ICD-10	Fracture of femur, part unspecified
S82	ICD-10	Fracture of lower leg, including ankle
S82.0	ICD-10	Fracture of patella
S82.1	ICD-10	Fracture of upper end of tibia
S82.2	ICD-10	Fracture of shaft of tibia
S82.3	ICD-10	Fracture of lower end of tibia
S82.4	ICD-10	Fracture of fibula alone
S82.5	ICD-10	Fracture of medial malleolus
S82.6	ICD-10	Fracture of lateral malleolus
S82.7	ICD-10	Multiple fractures of lower leg
S82.8	ICD-10	Fractures of other parts of lower leg
S82.9	ICD-10	Fracture of lower leg, part unspecified
S92	ICD-10	Fracture of foot, except ankle
S92.0	ICD-10	Fracture of calcaneus
S92.1	ICD-10	Fracture of talus
S92.2	ICD-10	Fracture of other tarsal bone(s)
S92.3	ICD-10	Fracture of metatarsal bone
S92.4	ICD-10	Fracture of great toe
S92.5	ICD-10	Fracture of other toe
S92.7	ICD-10	Multiple fractures of foot
S92.9	ICD-10	Fracture of foot, unspecified
V08	OPCS-4	Reduction of fracture of maxilla

V08.1	OPCS-4	Reduction of fracture of alveolus of maxilla
V08.2	OPCS-4	Open reduction of fracture of maxilla NEC
V08.3	OPCS-4	Closed reduction of fracture of maxilla NEC
V08.8	OPCS-4	Other specified
V08.9	OPCS-4	Unspecified
V09	OPCS-4	Reduction of fracture of other bone of face
V09.1	OPCS-4	Reduction of fracture of nasoethmoid complex of bones
V09.2	OPCS-4	Reduction of fracture of nasal bone NEC
V09.3	OPCS-4	Reduction of fracture of zygomatic complex of bones
V09.8	OPCS-4	Other specified
V09.9	OPCS-4	Unspecified
V11	OPCS-4	Fixation of bone of face
V11.1	OPCS-4	Intermaxillary fixation of maxilla
V11.2	OPCS-4	Internal fixation of maxilla NEC
V11.3	OPCS-4	Extraoral fixation of maxilla
V11.4	OPCS-4	Fixation of maxilla NEC
V11.5	OPCS-4	Removal of fixation from bone of face
V11.8	OPCS-4	Other specified
V11.9	OPCS-4	Unspecified
V15	OPCS-4	Reduction of fracture of mandible
V15.1	OPCS-4	Reduction of fracture of alveolus of mandible
V15.2	OPCS-4	Open reduction of fracture of mandible NEC
V15.3	OPCS-4	Closed reduction of fracture of mandible NEC
V15.8	OPCS-4	Other specified
V15.9	OPCS-4	Unspecified
V17	OPCS-4	Fixation of mandible
V17.1	OPCS-4	Intermaxillary fixation of mandible
V17.2	OPCS-4	Internal fixation of mandible NEC
V17.3	OPCS-4	Extraoral fixation of mandible
V17.4	OPCS-4	Removal of fixation from mandible
V17.8	OPCS-4	Other specified
V17.9	OPCS-4	Unspecified
V44	OPCS-4	Decompression of fracture of spine
V44.1	OPCS-4	Complex decompression of fracture of spine
V44.2	OPCS-4	Anterior decompression of fracture of spine
V44.3	OPCS-4	Posterior decompression of fracture of spine NEC
V44.4	OPCS-4	Vertebroplasty of fracture of spine
V44.5	OPCS-4	Balloon kyphoplasty of fracture of spine
V44.8	OPCS-4	Other specified
V44.9	OPCS-4	Unspecified
V45	OPCS-4	Other reduction of fracture of spine
V45.1	OPCS-4	Open reduction of fracture of spine and excision of facet of spine
V45.2	OPCS-4	Open reduction of fracture of spine NEC
V45.3	OPCS-4	Manipulative reduction of fracture of spine
V45.8	OPCS-4	Other specified
V45.9	OPCS-4	Unspecified

V46	OPCS-4	Fixation of fracture of spine
V46.1	OPCS-4	Fixation of fracture of spine using plate
V46.2	OPCS-4	Fixation of fracture of spine using Harrington rod
V46.3	OPCS-4	Fixation of fracture of spine using wire
V46.4	OPCS-4	Fixation of fracture of spine and skull traction HFQ
V46.5	OPCS-4	Removal of fixation device from spine
V46.8	OPCS-4	Other specified
V46.9	OPCS-4	Unspecified
W19	OPCS-4	Primary open reduction of fracture of bone and intramedullary fixation
W19.1	OPCS-4	Primary open reduction of fracture of neck of femur and open fixation using pin and plate
W19.2	OPCS-4	Primary open reduction of fracture of long bone and fixation using rigid nail NEC
W19.3	OPCS-4	Primary open reduction of fracture of long bone and fixation using flexible nail
W19.4	OPCS-4	Primary open reduction of fracture of small bone and fixation using screw
W19.5	OPCS-4	Primary open reduction of fragment of bone and fixation using screw
W19.6	OPCS-4	Primary open reduction of fragment of bone and fixation using wire system
W19.8	OPCS-4	Other specified
W19.9	OPCS-4	Unspecified
W20	OPCS-4	Primary open reduction of fracture of bone and extramedullary fixation
W20.1	OPCS-4	Primary open reduction of fracture of long bone and extramedullary fixation using plate NEC
W20.2	OPCS-4	Primary open reduction of fracture of long bone and extramedullary fixation using cerclage
W20.3	OPCS-4	Primary open reduction of fracture of long bone and extramedullary fixation using suture
W20.4	OPCS-4	Primary open reduction of fracture of long bone and complex extramedullary fixation NEC
W20.5	OPCS-4	Primary open reduction of fracture of ankle and extramedullary fixation NEC
W20.6	OPCS-4	Wiring of sternum
W20.8	OPCS-4	Other specified
W20.9	OPCS-4	Unspecified
W21	OPCS-4	Primary open reduction of intra-articular fracture of bone
W21.1	OPCS-4	Primary reduction of intra-articular fracture of bone using arthrotomy as approach
W21.2	OPCS-4	Primary excision of intra-articular fragment of intra-articular fracture of bone
W21.3	OPCS-4	Primary fixation of fragment of chondral cartilage of intra-articular fracture of bone
W21.4	OPCS-4	Primary intra-articular fixation of intra-articular fracture of bone NEC
W21.5	OPCS-4	Primary extra-articular reduction of intra-articular fracture of bone
W21.8	OPCS-4	Other specified
W21.9	OPCS-4	Unspecified
W22	OPCS-4	Other primary open reduction of fracture of bone
W22.1	OPCS-4	Primary open reduction of fracture of bone and skeletal traction HFQ
W22.2	OPCS-4	Primary open reduction of fracture of bone and external fixation HFQ
W22.8	OPCS-4	Other specified
W22.9	OPCS-4	Unspecified
W23	OPCS-4	Secondary open reduction of fracture of bone
W23.1	OPCS-4	Secondary open reduction of fracture of bone and intramedullary fixation HFQ
W23.2	OPCS-4	Secondary open reduction of fracture of bone and extramedullary fixation HFQ
W23.3	OPCS-4	Secondary open reduction of intra-articular fracture of bone
W23.4	OPCS-4	Secondary open reduction of fracture of bone and skeletal traction HFQ
W23.5	OPCS-4	Secondary open reduction of fracture of bone and external fixation HFQ
W23.6	OPCS-4	Secondary open reduction of fracture of bone and internal fixation HFQ

W23.8	OPCS-4	Other specified
W23.9	OPCS-4	Unspecified
W24	OPCS-4	Closed reduction of fracture of bone and internal fixation
W24.1	OPCS-4	Closed reduction of intracapsular fracture of neck of femur and fixation using nail or screw
W24.2	OPCS-4	Closed reduction of fracture of long bone and rigid internal fixation NEC
W24.3	OPCS-4	Closed reduction of fracture of long bone and flexible internal fixation HFQ
W24.4	OPCS-4	Closed reduction of fracture of small bone and fixation using screw
W24.5	OPCS-4	Closed reduction of fragment of bone and fixation using screw
W24.6	OPCS-4	Closed reduction of fracture of bone and fixation using nail or screw
W24.8	OPCS-4	Other specified
W24.9	OPCS-4	Unspecified
W25	OPCS-4	Closed reduction of fracture of bone and external fixation
W25.1	OPCS-4	Closed reduction of fracture of bone and fixation to skeleton HFQ
W25.2	OPCS-4	Closed reduction of fracture of bone and fixation using functional bracing system
W25.3	OPCS-4	Remanipulation of fracture of bone and external fixation HFQ
W25.8	OPCS-4	Other specified
W25.9	OPCS-4	Unspecified
W26	OPCS-4	Other closed reduction of fracture of bone
W26.1	OPCS-4	Manipulation of fracture of bone and skeletal traction NEC
W26.2	OPCS-4	Manipulation of fracture of bone NEC
W26.3	OPCS-4	Remanipulation of fracture of bone and skeletal traction NEC
W26.4	OPCS-4	Remanipulation of fracture of bone NEC
W26.8	OPCS-4	Other specified
W26.9	OPCS-4	Unspecified
W27	OPCS-4	Fixation of epiphysis
W27.1	OPCS-4	Permanent cross union epiphysiodesis
W27.2	OPCS-4	Epiphysioplasty
W27.3	OPCS-4	Insertion of staple into epiphysis
W27.4	OPCS-4	Removal of staple from epiphysis
W27.5	OPCS-4	Temporary fixation of epiphysis
W27.8	OPCS-4	Other specified
W27.9	OPCS-4	Unspecified
W28	OPCS-4	Other internal fixation of bone
W28.1	OPCS-4	Application of internal fixation to bone NEC
W28.2	OPCS-4	Adjustment to internal fixation of bone NEC
W28.3	OPCS-4	Removal of internal fixation from bone NEC
W28.4	OPCS-4	Insertion of intramedullary fixation and cementing of bone
W28.8	OPCS-4	Other specified
W28.9	OPCS-4	Unspecified
W29	OPCS-4	Skeletal traction of bone
W29.1	OPCS-4	Application of skeletal traction to bone NEC
W29.2	OPCS-4	Adjustment to skeletal traction of bone
W29.3	OPCS-4	Removal of skeletal traction from bone
W29.8	OPCS-4	Other specified
W29.9	OPCS-4	Unspecified
W30	OPCS-4	Other external fixation of bone

W30.1	OPCS-4	Application of external fixation to bone NEC
W30.2	OPCS-4	Adjustment to external fixation of bone NEC
W30.3	OPCS-4	Removal of external fixation from bone NEC
W30.4	OPCS-4	Application of external ring fixation to bone NEC
W30.8	OPCS-4	Other specified
W30.9	OPCS-4	Unspecified
W65.1	OPCS-4	Primary open reduction of fracture dislocation of joint and skeletal traction HFQ
W65.3	OPCS-4	Primary open reduction of fracture dislocation of joint NEC
W65.4	OPCS-4	Primary open reduction of fracture dislocation of joint and internal fixation NEC
W65.5	OPCS-4	Primary open reduction of fracture dislocation of joint and combined internal and external fixation
W66.1	OPCS-4	Primary closed reduction of fracture dislocation of joint and skeletal traction HFQ
W66.3	OPCS-4	Primary manipulative closed reduction of fracture dislocation of joint NEC
W66.4	OPCS-4	Primary closed reduction of fracture dislocation of joint and internal fixation
W67.1	OPCS-4	Secondary open reduction of fracture dislocation of joint and skeletal traction HFQ
W67.3	OPCS-4	Secondary open reduction of fracture dislocation of joint NEC
W67.5	OPCS-4	Remanipulation of fracture dislocation of joint
W67.7	OPCS-4	Secondary open reduction of fracture dislocation of joint and internal fixation NEC
X48	OPCS-4	Immobilisation using plaster cast
X48.1	OPCS-4	Application of plaster cast
X48.2	OPCS-4	Change of plaster cast
X48.3	OPCS-4	Removal of plaster cast
X48.8	OPCS-4	Other specified
X48.9	OPCS-4	Unspecified
X49	OPCS-4	Other external support of limb
X49.1	OPCS-4	Application of splint NEC
X49.2	OPCS-4	Change of splint NEC
X49.3	OPCS-4	Removal of splint NEC
X49.4	OPCS-4	Skin traction
X49.5	OPCS-4	Application of sling NEC
X49.6	OPCS-4	Application of elastic support bandage NEC
X49.7	OPCS-4	Application of gauze support bandage NEC
X49.8	OPCS-4	Other specified
X49.9	OPCS-4	Unspecified

Web Table 2. Included opioid drugs and equianalgesic ratios

Opioid Drug, Source	Form[a]	Equianalgesic Ratio
Alfentanil,[4]	SPR	30.00
Buprenorphine,[2]	TD	110.00
	OD	50.00
Codeine,[7]		0.15
Dextromoramide,[3]		2.00
Dextropropoxyphene,[7]		0.15
Diamorphine[b]		1.00
Dihydrocodeine,[7]		0.13
Dipipanone,[5]		0.50
Fentanyl,[7,3,5,1]	TD	100.00
	OD	50.00
	SPR	160.00
Hydromorphone,[7]		6.00
Methadone,[2]		3.00
Meptazinol,[5]		0.03
Morphine,[7]		1.00
Oxycodone,[7]		1.50
Pentazocine,[1]		0.37
Pethidine,[7]		0.10
Tapentadol,[2]		0.40
Tramadol,[7]		0.20

Abbreviations: SPR, sprays (buccal and nasal); TD, transdermal patch; OD, orodispersible.

[a] form is an oral preparation unless otherwise stated.

[b] rarely prescribed as an oral formulation; equianalgesic ratio based on advice from a specialist pain management pharmacist.

Web Figure 1. Overview of opioid prescription preparation process[a]

		Setting values	Generating variables	Identifying records	Imputing/handling values	Removing records
Cleaning	Quantity and dose	Minimum and maximum quantity and daily dose		Missing and implausible quantities	Missing and implausible quantities in a series of steps[b]	Patients with any remaining missing or implausible quantities
				Missing and implausible doses	Missing and implausible doses in a series of steps[b]	Patients with any remaining missing or implausible doses
Duration	Duration and stop date	Maximum value for duration	Duration based on quantity and dose; stop date based on start date and duration	Records with multiple durations	Replace with mean of durations if ≤30 days apart (those >30 set to missing)	
				Missing and implausible durations	Missing and implausible durations via a series of steps[c]	
Gap and Overlaps	Overlapping identical products			Records for identical products with the same start date	Replace duration with the sum of durations	Excess records after combining
				Records with overlapping days	Start and stop dates, and durations for overlapping days moved to gaps and end of records	Overlapping days that extended beyond end of follow-up
	Gaps between identical products	Permissible gap		Records for identical products that have a permissible gap (<15 days)	Stop date and duration extended to close the permissible gap	
OMEQ dose	OMEQ dose/day	Equianalgesic ratio	OMEQ dose/day for each record			
	Total OMEQ dose/day		Opioid exposure status (yes/no)	Overlapping records for different products	Summed OMEQ dose/day for overlapping days	Join records for any opioid into continuous exposed periods and drop excess records

[Figure legend on following page]

Abbreviations: OMEQ, oral morphine equivalent.

[a] adaptation and extension of the DrugPrep framework and respective Stata code published by Pye et al. (2018).[6]

[b] step 1: the value (quantity and/or daily dose) was replaced with the value recorded for a subsequent prescription for the same product, for the same patient. If there was no subsequent prescription for the product, or if the value for the subsequent prescription was missing or implausible, step 2 was followed. Step 2: the value was replaced with the value recorded for the previous prescription for the same product, for the same patient. If there was no previous prescription for the product, or if the value on the previous prescription was missing or implausible, step 3 was followed. Step 3: the value was replaced with the median value for the individual patient, taken from all plausible values recorded for their prescriptions for the same product. If there were no other prescriptions for the product, or if the values recorded for all other prescriptions were also missing or implausible, step 4 was followed. Step 4: the value was replaced with the population-median value, taken from all plausible values recorded for all prescriptions for the same product, across all patients in the study cohort. If there were no other prescriptions for the product, or if the values recorded on all other prescriptions were also missing or implausible, these records were removed, as detailed in the following section.

[c] step 1: the 'new duration' was replaced using the median duration for the individual patient, taken from all of their prescriptions for the same product. If there were no other prescriptions for the product, or if the durations recorded on all other prescriptions were also missing, step 2 was followed. Step 2: the 'new duration' was replaced using the population-median duration, taken from all prescriptions for the same product, across the entire study cohort.

Web Figure 2. Proximity of fracture events to opioid initiation and definition of the pre-exposure risk period

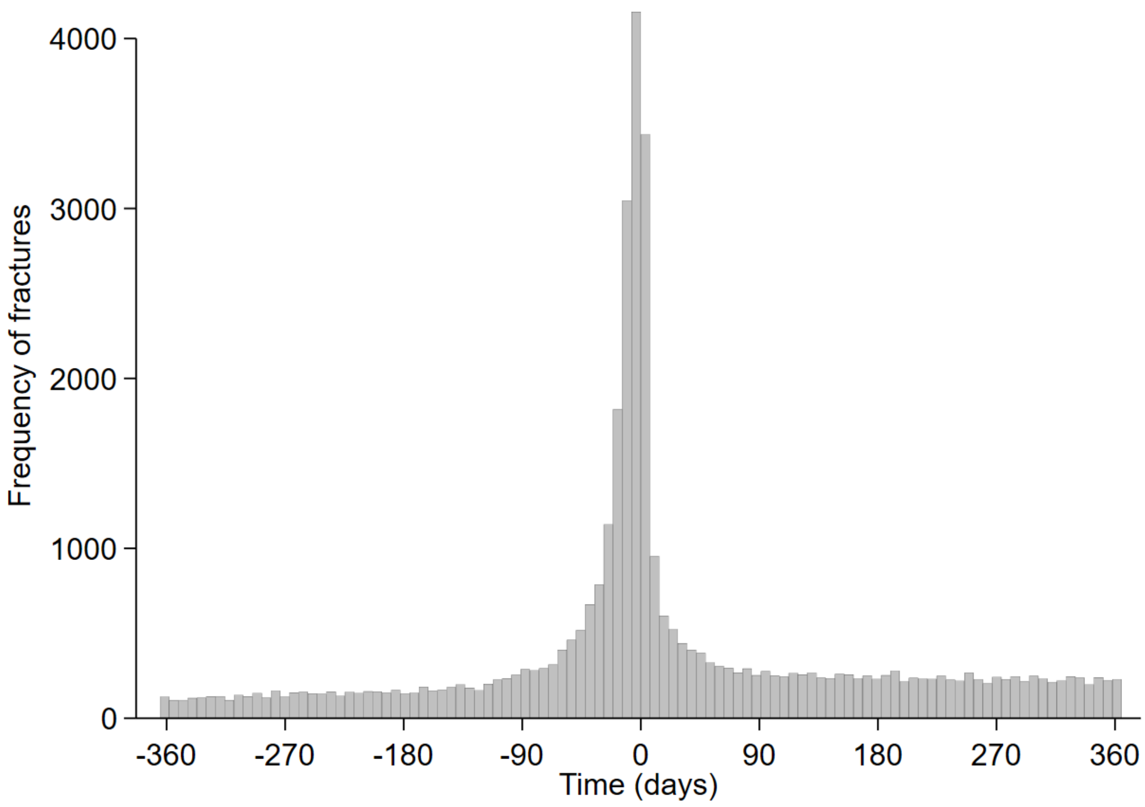

Notes: Time-point 0 indicates that the date of fracture and opioid initiation were the same. A positive value indicates that a fracture occurred after opioid initiation, and a negative value indicates that a fracture occurred before opioid initiation. The rate of fracture stabilizes >90 days before opioid initiation which indicated that 90 days was an appropriate duration for the pre-exposure risk period.

Web Figure 3. Curtailment of overlapping risk periods

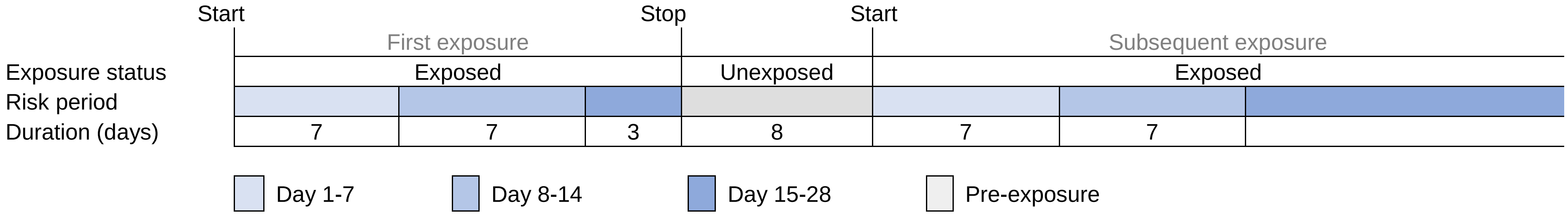

Notes: In this example the risk period ’Day 15-28’ for the first exposed period, which is ordinarily 14 days in duration was curtailed at three days due to the stop date of the period of exposure. An eight day gap followed which was too short to incorporate a 28-day post-exposure period, baseline period and 90-day pre-exposure period, therefore the pre-exposure period took priority and was curtailed to eight days due to the restart of an opioid.

Web Table 3. Fracture risk increasing drugs (excluding opioids)

**Psychoactive Drugs**				
agomelatine	co-beneldopa	levetiracetam	pericyazine	sertraline
alprazolam	co-careldopa	levopromazine	perphenazine	sodium oxybate
amantadine	diazepam	lithium	phenelzine	sodium valproate
amisulpiride	dosulepin	lofepramine	phenobarbital	sulpiride
amitriptyline	doxepin	loprazolam	phenytoin	temazepam
apomorphine	duloxetine	lorazepam	pimozide	thiopental
aripiprazole	entacapone	lorazepam	pramipexole	tigabine
asenapine	escitalopram	lormetazepam	pregabalin	tolcapone
benperidol	eslicarbazepine	melatonin	primidone	topiramate
bromocriptine	ethosuximide	meprobamate	prochlorperazine	tranylcypromine
buspirone	fluoxetine	mianserin	procyclidine	trazodone
cabergoline	flupentixol	mirtazapine	promazine	trifluoperazine
carbamazepine	flupentixol	moclobemide	promethazine	trihexyphenidyl
chloral hydrate	fluphenazine	nitrazepam	quetiapine	trimipramine
chlordiazepoxide	flurazepam	nortriptyline	rasagiline	valproic acid
chlorpromazine	fluvoxamine	olanzapine	reboxetine	venlafaxine
citalopram	gabapentin	orphenadrine	retigabine	vigabatrin
clobazam	haloperidol	oxycarbazepine	risperidone	zaleplon
clomipramine	imipramine	paliperidone	ropinerole	zolpidem
clomthiazole	isocerboxazid	paroxetine	rotigotine	zonisamide
clonazepam	lacosamide	perampanel	rufinamide	zopiclone
clozapine	lamotrigine	pergolide	selegiline	zuclopentixol

**Cardiovascular Drugs**				
acebutolol	cilazapril	frusene	metoprolol	riociguat
aliskiren	clonidine	furosemide	minoxidil	sildenafil
ambrisentan	co-amilofrise	hydralazine	moexipril	sodium nitroprusside
amiloride	co-amilozide	iloprost	moxonidine	sotalol
atenolol	co-flumactone	imidapril	nadolol	spironolactone
azilsartan	co-triamterzide	indapamide	nebivolol	tadalafil
bendroflumethiazide	cyclopenthiazide	indoramin	olmesartan	telmisartan
bisoprolol	digoxin	irbesartan	oxprenolol	terazosin
bosentan	doxazosin	labetalol	perindopril	timolol
bumetanide	enalapril	lasilactone	phenoxybenzamine	torasemide
candesartan	eplerenone	lisinopril	phentolamine	trandolapril
captopril	eprosartan	losartan	prazosin	triamterene
carbedilol	esmolol	macitentan	propanolol	valsartan
celiprolol	flecainide	methyldopa	quinapril	xipamide
chlortalidone	fosinopril	metolazone	ramipril	

**Steroid and Glucocorticoid Drugs**				
betamethasone	dexamethasone	methylprednisolone	prednisone	
deflazacort	hydrocortisone	prednisolone		

Web Figure 4. Selection of study cohort

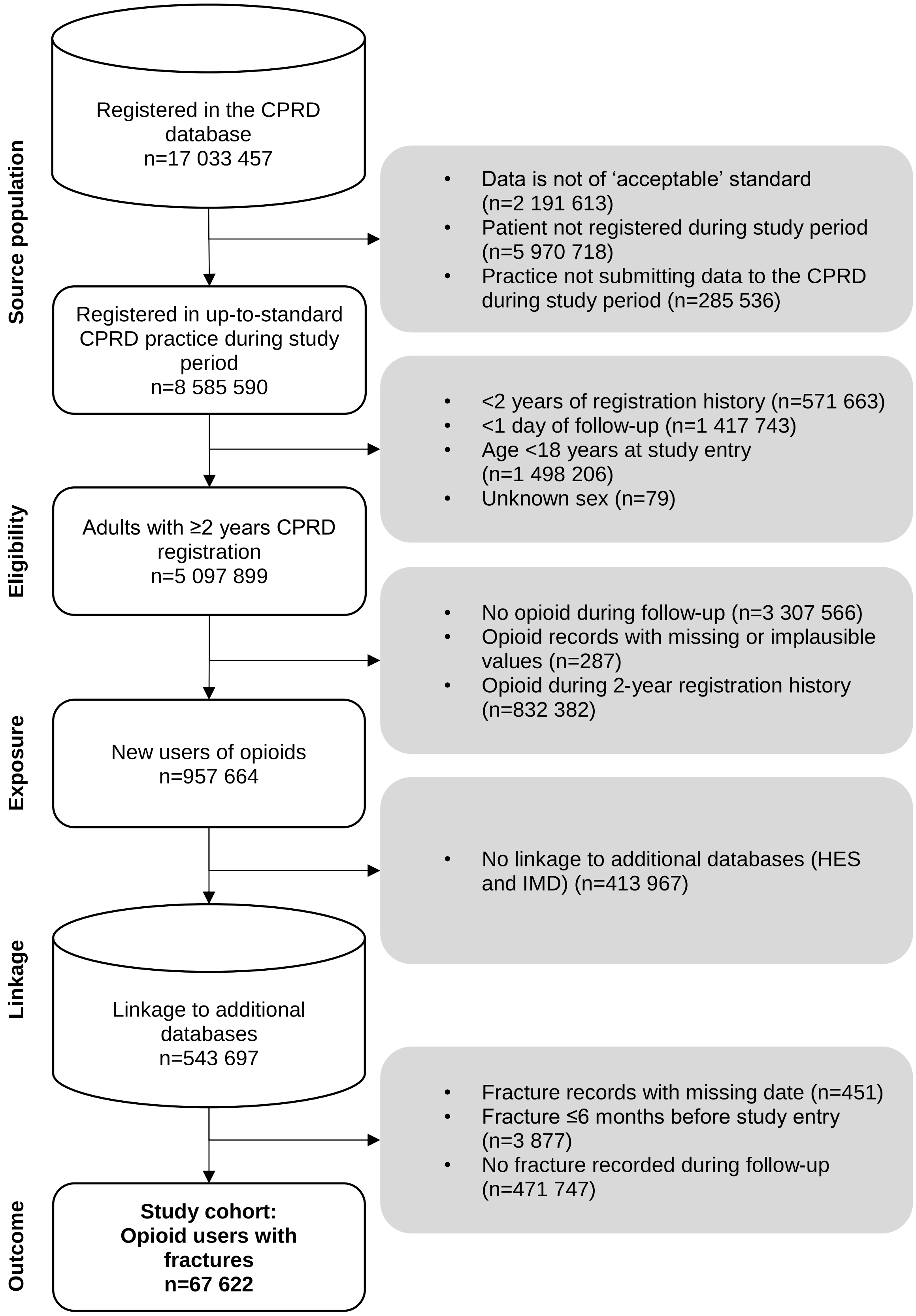

Abbreviations: CPRD, Clinical Practice Research Datalink; HES, Hospital Episode Statistics; IMD, Index of Multiple Deprivation.

Notes: Study period from June 1 2008, to May 31 2017.

Web Table 4. Sensitivity analyses

	1[a]	2[b]	3[c]	4[d]	5[e]	6[f]	7[g]
Risk period	aIRR[h] (95% CI)	aIRR[h] (95% CI)	aIRR[h] (95% CI)	aIRR[h] (95% CI)	aIRR[h] (95% CI)	aIRR[h] (95% CI)	aIRR[h] (95% CI)
Baseline	1 [Reference]	1 [Reference]	1 [Reference]	1 [Reference]	1 [Reference]	1 [Reference]	1 [Reference]
Pre-exposure	5.53 (5.44-5.62)	5.81 (5.71-5.92)	5.68 (5.55-5.82)	4.88 (4.75-5.01)	6.01 (5.89-6.13)	12.85 (12.55-13.16)	9.65 (9.48-9.83)
Post-exposure	2.25 (2.16-2.34)	2.27 (2.17-2.38)	2.26 (2.13-2.40)	2.36 (2.22-2.51)	2.21 (2.11-2.33)	1.85 (1.79-1.91)	2.25 (2.17-2.33)
First exposure							
Days 1-7	7.73 (7.31-8.17)	7.74 (7.28-8.24)	7.95 (7.37-8.57)	6.92 (6.31-7.59)	8.00 (7.49-8.54)	6.10 (5.77-6.44)	7.18 (6.80-7.59)
Days 8-14	5.08 (4.68-5.51)	4.90 (4.46-5.37)	4.96 (4.43-5.56)	4.34 (3.78-4.99)	4.89 (4.42-5.41)	3.96 (3.65-4.29)	4.68 (4.31-5.07)
Days 15-28	3.60 (3.17-4.08)	3.61 (3.13-4.16)	3.39 (2.82-4.07)	2.71 (2.17-3.38)	3.48 (2.97-4.07)	2.79 (2.47-3.16)	3.37 (2.98-3.81)
Days 29-365	1.74 (1.51-2.01)	1.80 (1.53-2.12)	1.67 (1.34-2.09)	1.74 (1.42-2.14)	1.58 (1.32-1.90)	1.33 (1.15-1.53)	1.64 (1.43-1.89)
Day 366+	1.34 (0.92-1.96)	1.07 (0.66-1.74)	1.47 (0.83-2.61)	1.17 (0.69-1.98)	1.26 (0.81-1.96)	0.92 (0.63-1.34)	1.15 (0.79-1.68)
Subsequent exposures							
Days 1-7	4.80 (4.58-5.04)	5.13 (4.86-5.42)	4.74 (4.32-5.20)	4.77 (4.43-5.14)	4.77 (4.48-5.08)	3.58 (3.42-3.74)	4.84 (4.62-5.06)
Days 8-14	3.56 (3.34-3.79)	3.75 (3.49-4.04)	3.04 (2.66-3.47)	3.49 (3.16-3.84)	3.58 (3.29-3.88)	2.62 (2.47-2.79)	3.57 (3.36-3.80)
Days 15-28	3.04 (2.82-3.27)	3.06 (2.80-3.34)	3.04 (2.61-3.53)	2.84 (2.53-3.19)	2.83 (2.56-3.13)	2.21 (2.06-2.38)	3.04 (2.83-3.26)
Days 29-365	2.37 (2.24-2.50)	2.31 (2.16-2.48)	2.12 (1.86-2.38)	2.40 (2.20-2.62)	2.46 (2.29-2.65)	1.73 (1.64-1.83)	2.40 (2.27-2.53)
Day 366+	1.73 (1.51-1.99)	1.61 (1.35-1.93)	1.27 (0.95-1.70)	1.73 (1.39-2.15)	1.75 (1.47-2.08)	1.21 (1.05-1.38)	1.70 (1.48-1.95)

Abbreviations: aIRR, adjusted incidence rate ratio; CI, confidence interval.
[a] excluding patients that died ≤90 days after first fracture.
[b] outcome defined as first fractures only.
[c] excluding patients that had dose or duration data imputed i.e., complete-case analysis.
[d] excluding patients with fractures identified in the CPRD database.
[e] excluding patients with cancer recorded (ever) in the CPRD and HES databases.
[f] 7-day duration for pre-exposure risk period.
[g] 28-day duration for pre-exposure risk period.
[h] adjusted for 1-year increments in age, 3-monthly intervals for season.
Notes: aIRRs for the pre-exposure risk period are based on the fracture rate in the 90-day period prior to, and including the first day of opioid exposure, compared to the baseline rate of fracture. Pre-exposure aIRRs are likely influenced by opioid prescribing in response to fracture, resulting in greater aIRRs when compared to the baseline risk period.

Web Figure 5. Risk of fracture when exposed to opioids by anatomical site

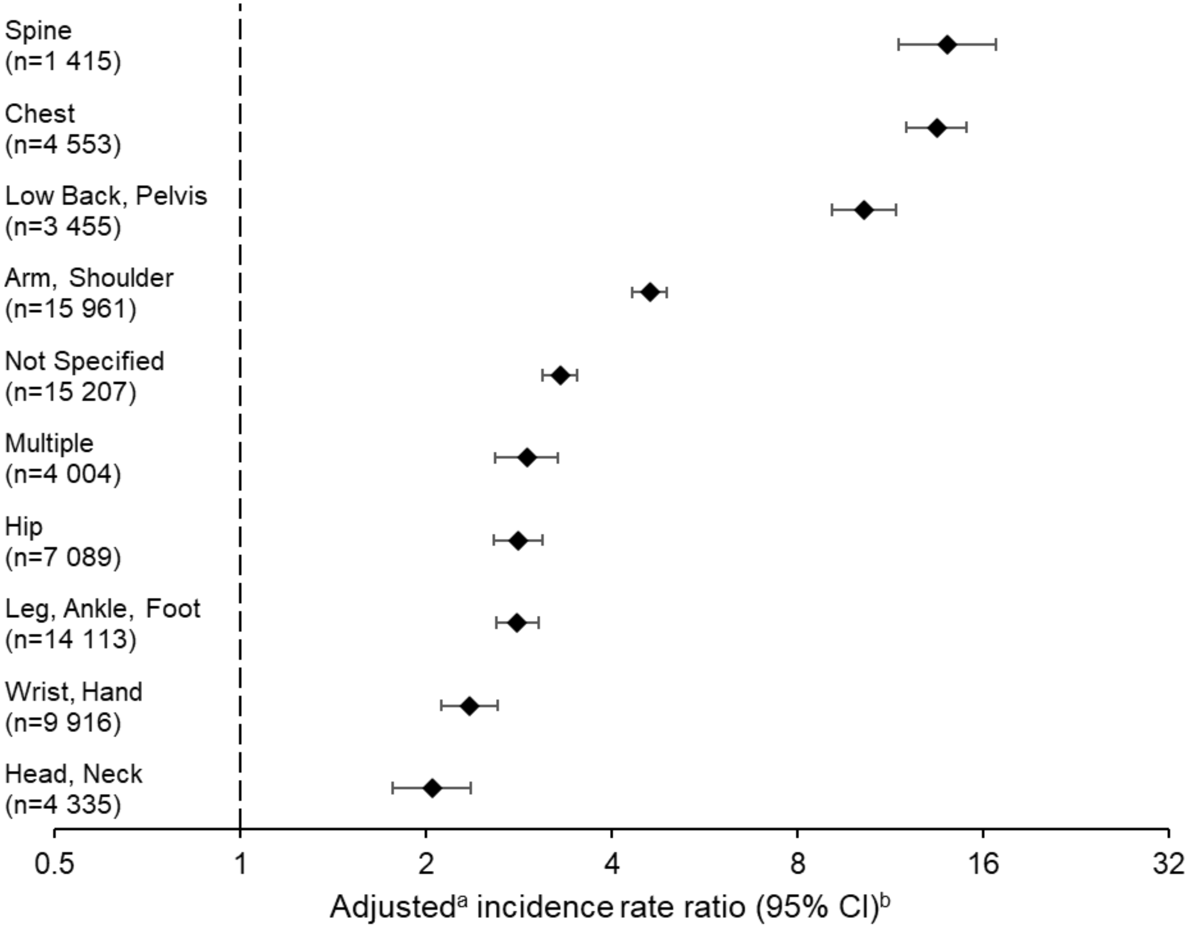

[a] adjusted for 3-year increments in age and 3-monthly intervals for season.

[b] values plotted on logarithmic scale.

Web Figure 6. Comparison of aIRRs in primary analysis and after excluding fractures to spine, chest, low back and pelvis

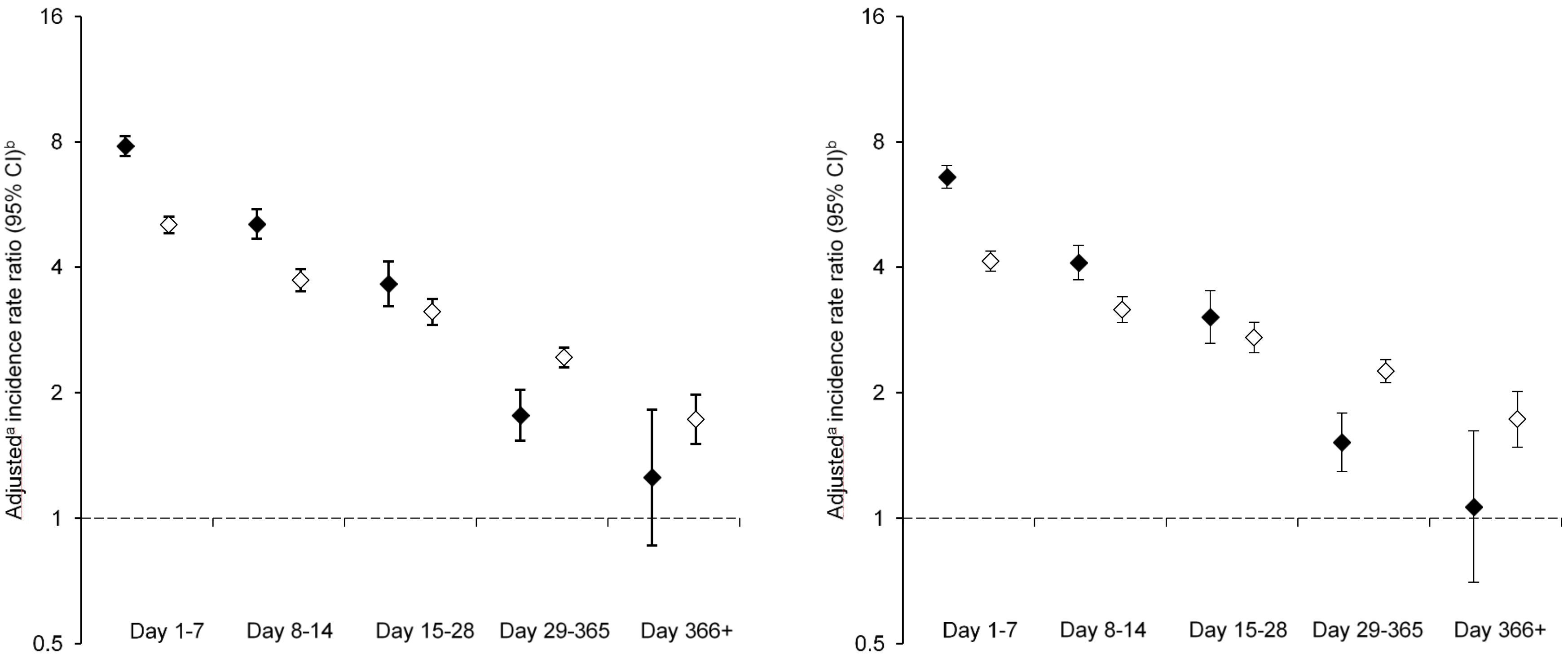

(a) Includes all fracture sites **(b) Excludes fractures to spine, chest, low back and pelvis**

Notes: black diamonds refer to aIRRs for the first exposure; hollow diamonds refer to aIRRs for subsequent exposure periods.

[a] adjusted for 1-year increments in age and 3-monthly intervals for season.

[b] values plotted on logarithmic scale.

Web Figure 7. Risk of falls when exposed to opioids

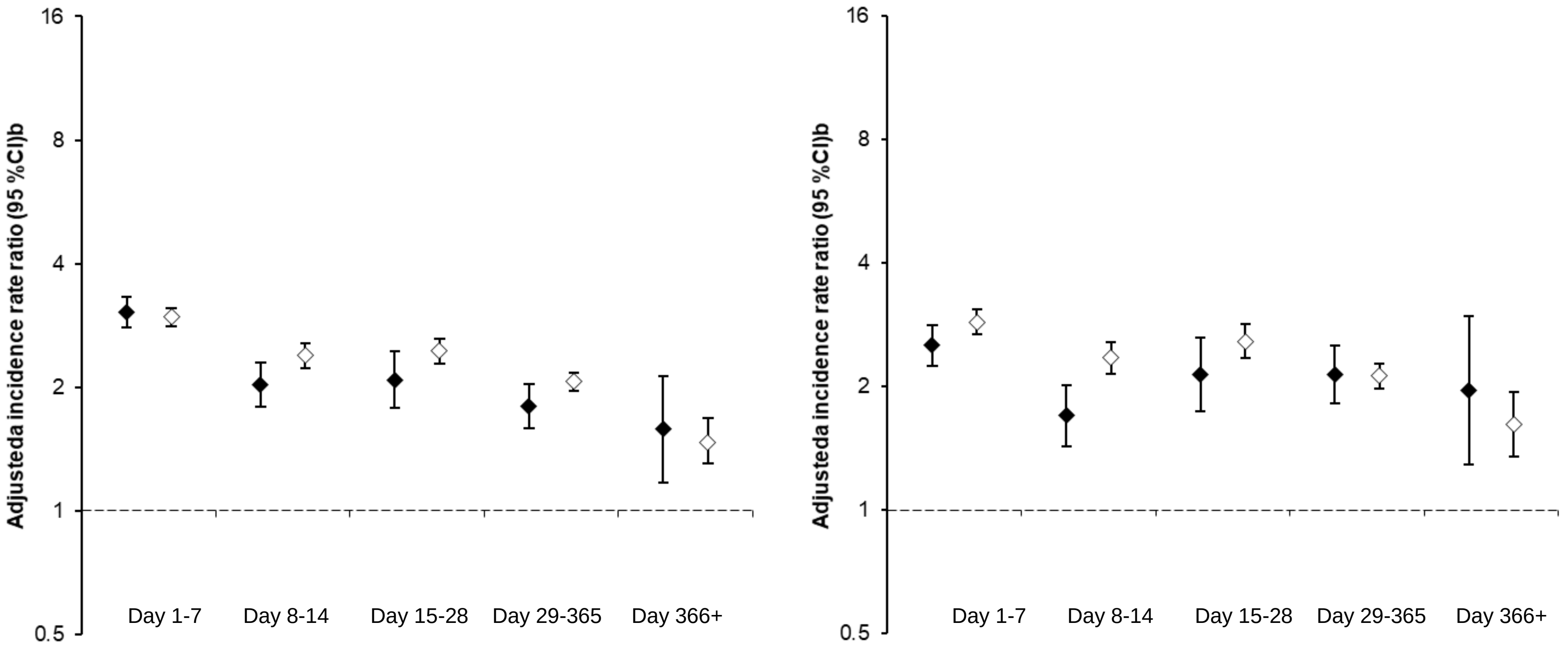

(a) Includes individuals who also sustained fractures (n=58 774) **(b) Excludes individuals who sustained fractures (n=38 756)**

Notes: black diamonds refer to aIRRs for the first exposure; hollow diamonds refer to aIRRs for subsequent exposure periods.

[a] adjusted for 1-year increments in age and 3-monthly intervals for season.

[b] values plotted on logarithmic scale.

References

[1] Centers for Medicare & Medicaid Services. Opioid Oral Morphine Milligram Equivalent (MME) Conversion Factors, 2017.

[2] Els C, Jackson TD, Kunyk D, Lappi VG, Sonnenberg B, Hagtvedt R, Sharma S, Kolahdooz F, Straube S. Adverse events associated with medium- and long-term use of opioids for chronic non-cancer pain: an overview of Cochrane Reviews. Cochrane Database Syst Rev 2017;10:Cd012509.

[3] Keats AS, Telford J, Kurosu Y. Studies of analgesic drugs: III. Dextromoramide and a comparison of methods of estimating pain relief in man. J Pharmacol Exp Ther 1960;130(2):212.

[4] National Health Service (NHS) Scotland. Scottish Palliative Care Guidelines, 2019.

[5] National Health Service (NHS) Wales. Opiate Conversion Doses, 2010.

[6] Pye SR, Sheppard T, Joseph RM, Lunt M, Girard N, Haas JS, Bates DW, Buckeridge DL, van Staa TP, Tamblyn R, Dixon WG. Assumptions made when preparing drug exposure data for analysis have an impact on results: An unreported step in pharmacoepidemiology studies. Pharmacoepidemiol Drug Saf 2018;27(7):781-788.

[7] Svendsen K, Borchgrevink P, Fredheim O, Hamunen K, Mellbye A, Dale O. Choosing the unit of measurement counts: the use of oral morphine equivalents in studies of opioid consumption is a useful addition to defined daily doses. Palliat Med 2011;25(7):725-732.
